# tRNA modifications in viral replication

**DOI:** 10.1016/j.jbc.2026.111430

**Published:** 2026-04-07

**Authors:** Chathuri Pathirage, Kristin S. Koutmou, Karin Musier-Forsyth

**Affiliations:** 1 Department of Chemistry, University of Michigan, Ann Arbor, Michigan, USA; 2 Department of Chemistry and Biochemistry, Center for Retrovirus Research, and Center for RNA Biology, Ohio State University, Columbus, Ohio, USA

**Keywords:** anti-viral immune response, codon usage, reverse transcription, RNA modification, transfer RNA (tRNA), translation regulation, tRNA-derived RNA (tDR), tRNA-like structures (TLS), tRNA-modifying enzymes, viral replication

## Abstract

tRNA modifications are key players in post-transcriptional gene expression regulation under external and cellular stresses. Viral infection is a common external stress that hijacks cellular processes for replication. Host cell tRNA pools and modification profiles are often reshaped during viral infection, eliciting both pro- and anti-viral effects. Changes in the host tRNA modifications, particularly in the anticodon sequence, have the capability to reprogram host and viral proteomes. For example, anticodon loop modifications contribute to programmed ribosomal frameshifting essential for producing certain viral proteins. However, the roles of tRNA modifications are not limited to translation during viral infection. Retroviruses use select host cell tRNAs as reverse transcription primers, and modifications modulate steps in reverse transcription. Furthermore, some non-primer modified tRNAs are selectively packaged into virion particles, though their functions remain unknown. Virally encoded tRNAs harbor modifications that expand the anticodon pool, as host tRNAs are depleted. Expression and activity of several tRNA-modifying enzymes are regulated upon viral infection, the functional implications of which remain to be elucidated. tRNA modifications are involved in anti-viral defense, particularly in tRNA cleavage at the anticodon loop following viral infection, leading to tRNA-derived fragments. While the tRNA modification landscape is likely significantly altered following most viral infections, current evidence is limited to a few specific examples. A global tRNAome analysis during viral infection will shed light on the regulation of these and other processes. Emerging technologies, including advances in direct tRNA sequencing and modification detection *via* mass spectrometry, are making this possible.

tRNAs are abundant and essential biomolecules present in all living organisms. They are primarily responsible for delivering cognate amino acids to the ribosome in accordance with mRNA codons during protein synthesis. Beyond this fundamental and critical function, tRNAs serve a variety of regulatory roles, including eliciting stress responses, regulating global gene expression, and modulating apoptosis, underscoring their broader importance in cellular metabolism and homeostasis (1, 2, 3).

tRNAs fold into a stable cloverleaf secondary structure and an L-shaped tertiary structure, stabilized through Watson-Crick base-pairing and long-range interactions, respectively. tRNAs contain four stem or stemloop structures – the anticodon (AC) stemloop, dihydrouridine (D) stemloop, T or TψC stem loop, and the acceptor stem (Fig. 1*A*) (4, 5). While preserving this common structure, tRNAs maintain their individuality through specific identity elements that are recognized by the cognate aminoacyl-tRNA synthetases – the enzymes that link the correct amino acid to the 3′-end of the tRNA. In the ribosome, nucleotides (nt) 34 to 36 in the AC loop of the tRNA base pair with the mRNA codon. Nucleotide 34 is designated the “wobble” position as it often base-pairs with the third nt of the codon in a non-Watson-Crick manner. This allows maintenance of the degeneracy of the genetic code, where the 61 sense codons coding for the 20 amino acids can be deciphered by a limited number of tRNAs (6, 7).

**Figure 1:**
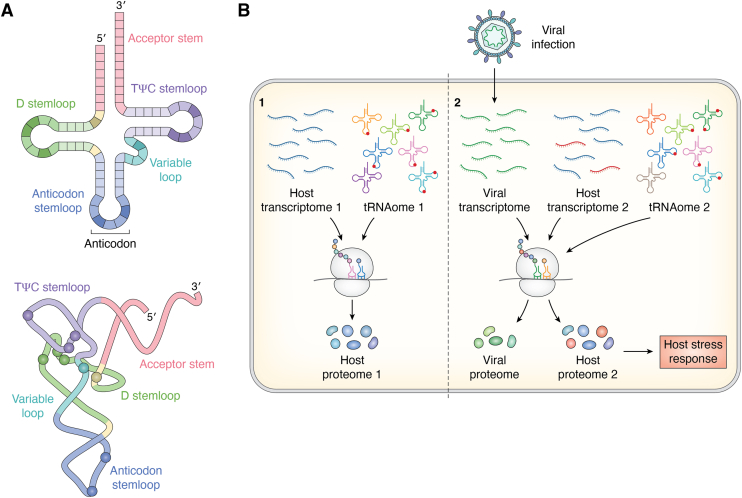
**Viral infection alters host tRNA pools and modification profiles.***A*, secondary and tertiary structure of tRNA. tRNA folds into a cloverleaf secondary structure (*top*) with acceptor stem, TψC stemloop, D stemloop, variable loop and the anticodon stemloop, and an L-shaped tertiary fold (*bottom*). Spheres indicate commonly modified nucleotide (nt) positions: nt 9, 16, 17 and 20 in D loop, nt 34 (wobble position) and 37 in anticodon loop, nt 47 in the variable loop and nt 54, 55 and 58 in the TψC loop. *B*, changes in the host transcriptome and proteome upon viral infection 1 (*left*). The host mRNA transcriptome (1), tRNAome (1) (tRNA abundance, modifications and fragmentation) and the proteome (1) in uninfected cells 2 (*right*). Viral infection adjusts the modified tRNA pool (tRNAome 2) through either host or viral tRNA modifying enzymes. The changes in the tRNA modification profile, particularly at the AC, may facilitate translation of viral proteins, either by matching viral codon-usage or promoting viral PRF. These changes in the tRNA epitranscriptome could also generate a host stress response to infection (host proteome 2), affecting tRNA stability and triggering tRNA degradation mechanisms. Viral infection may also alter modification profiles to ensure specific tRNAs are well suited to perform functions related to viral replication such as serving as reverse transcription primers.

As the most highly decorated nucleic acid species, tRNAs contain eight to 13 posttranscriptional RNA modifications per molecule, on average (8). Approximately 80% of all known RNA modifications are found in tRNAs. Modifications facilitate the main role of tRNAs as adapter molecules in translation as well as their moonlighting functions such as stress response and adaptation, metabolite modulation, regulation of cell cycle and aging mechanisms, immune signaling and pathological regulation (9, 10, 11, 12, 13, 14). The AC loop is a hotspot for modifications, with positions 32, 34, and 37 to 39 being most commonly modified. Modifications at the wobble position maintain the degeneracy of the genetic code, while those at position 37 stabilize codon: anticodon interactions and maintain the correct reading-frame (12, 15). Modifications in the tRNA body are important for folding into the correct structure, cellular stability, accurate aminoacylation, fine-tuning translation rate and tRNA-derived RNA (tDR) biogenesis (12, 16). A handful of tRNA modifications are reversible, and the pool of cellular tRNAs is in flux (17, 18, 19). This allows cells to fine-tune regulatory processes in response to external or internal stimuli such as temperature shifts, nutrient availability, and stress, or processes during development, differentiation, and cell death (17, 18, 20, 21, 22). In higher organisms such as humans, tRNA modifications show tissue-specific enrichment patterns (23). Abnormalities in tRNA modification profiles have been linked to many diseases, including cancer and neurological, developmental, mitochondrial, and autoimmune disorders (12).

While the tRNAome is well established as a dynamic modulator of gene expression (24, 25, 26), the function of tRNAs as regulators in viral infection is also starting to be acknowledged. This is both due to their indispensable role in cellular metabolism and homeostasis, as well as specific functions bestowed upon them by viral or host systems during infection. While roles of specific tRNA modifications during viral infection have been known for some time, the global changes in the tRNA epitranscriptome and their biological significance are only beginning to emerge, owing to advances in modification detection and deep sequencing tools (27, 28, 29, 30, 31, 32).

In this review, we summarize current understanding of how the host tRNA modification landscape is altered during viral infection and how these changes impact global protein synthesis, including viral protein production *via* programmed ribosomal frameshifting (PRF) events. The review reports known modifications found in tRNAs packaged into virus particles, their potential influence on the mechanisms of packaging, and their roles in tRNAs that serve as primers during retroviral replication. Furthermore, we discuss the regulation of tRNA-modifying enzymes as well as potential functions of modifications on viral tRNAs and tRNA-like structures (TLSs). This review also describes instances where tRNA modifications are used in anti-viral defense mechanisms. Finally, we highlight the major open questions in the field and envision how the development and application of new tools related to tRNA sequencing and modification detection will accelerate progress towards understanding the complex interplay between viral infection and tRNA modifications.

## Viral infections alter the tRNA epitranscriptome and impact protein synthesis

Cells regulate components of the translational machinery, including ribosomes, translation factors, tRNAs, amino acid levels, and mRNA expression and stability, to bring about suitable responses to many environmental and cellular changes at the transcriptomic and proteomic levels (33, 34, 35, 36, 37, 38, 39, 40, 41). As obligate intracellular parasites dependent on the host translation machinery to synthesize viral proteins, viruses have evolved many strategies to take control of the host translation system (42, 43). In response, cells co-evolved mechanisms to regulate protein synthesis, to provoke stress responses and anti-viral defenses. Thus, an arms race exists between viruses and their hosts (8, 44, 45, 46). The following three sections summarize current knowledge on the impact of viral infection on the global tRNA epitranscriptome and the effects of these changes on cellular and viral protein synthesis.

### tRNA reprogramming and viral codon usage

The redundancy of the genetic code means that different synonymous codons exist for the same amino acid. However, organisms frequently preferentially utilize certain synonymous codons over others, exhibiting a codon bias in their genes and/or genomes. This bias is correlated to the dynamic tRNA pools in cells; tRNAs decoding optimal or preferred codons are more abundant than those that decode rare codons (47, 48). Some viral genomes display a codon usage that matches the host. Bioinformatics studies demonstrated that some viruses that infect bacterial systems have fully adapted to their hosts' codon usage (49, 50, 51). However, other viruses have a different codon usage than their host systems. For example, human mRNAs are enriched in G/C-ending codons, while dengue virus (DENV), severe acute respiratory syndrome coronavirus-2 (SARS-CoV-2) and chikungunya virus (CHIKV) are enriched in A/U-ending codons, human T-lymphotropic virus-1 (HTLV-1) prefers C-rich codons, and lentiviruses, including human immunodeficiency virus type 1 (HIV-1) are enriched in A-ending codons (52, 53, 54, 55, 56, 57, 58, 59, 60). DENV was shown to preferentially use human and mosquito non-optimal codons. During DENV infection, human genes that use those non-optimal codons were also upregulated (61). Adding to this complexity, some viral genes show codon usage differences based on their temporal expression; in HIV-1, early-expressed genes (*tat*, *rev* ,and *nef*) use codons that are similar to those in highly-expressed host genes, while late genes (*gag*, *pol*, *env*, *vif*, *vpu*, and *vpr*) exhibit a rare codon usage pattern (53). A recent study showed that respiratory syncytial virus (RSV) infection causes ribosome recruitment and enhanced translation of virus-resembling AU-rich host transcripts, suggesting virus-induced changes to the overall translational landscape (62).

Changes in tRNA abundance are observed upon infection of several viruses (55, 63, 64, 65) (Fig. 1*B*). Following infection by DNA tumor viruses, an increase in global tRNA or pre-tRNA expression through RNA polymerase III activity has been reported (66, 67). In contrast, no changes in the total tRNA pool were observed upon vaccinia and influenza A infection. Instead, the tRNA populations associated with polysomes shifted to match the viral codon usage (68).

An *in silico* study on the relative codon usage landscape of 502 human-infecting viruses demonstrated that viruses with distinct tissue tropisms show codon usage adaptation to the tRNA abundances of the tissues they infect (69). This study compared tRNA abundance data across 23 human tissues from The Cancer Genome Atlas and identified that SARS-CoV-2 expression is specifically adapted to the upper respiratory tract and alveoli (69). Codon usage of early viral protein genes is better adapted to the tissue-specific tRNA pool than late protein genes; a reprogramming of the host tRNA landscape to match late viral protein expression is likely. A caveat of this study is that the tRNA abundance data is obtained from cancer cells, where dysregulation of tRNA expression is known to occur and thus may not represent the tRNA pools in healthy counterparts (57, 70). In another study, HIV-1 genes showed a strong correlation with the codon usage in human monocytes compared to B or T lymphocytes, which has implications for how the monocyte reservoir is established and maintained (52).

Host cell responses to viral infection can also reshape the cellular tRNA pool. For example, during Hepatitis E virus (HEV) infection, remodeling of the tRNA pool leads to activation of the inflammasome (71). Similarly, in certain retroviral infections such as HIV-1, Schlafen (SLFN) proteins, an interferon-stimulated family of early response genes, act as host restriction factors that counteract virus-induced changes in the tRNA pool. This is mainly accomplished by SLFN proteins binding to and degrading tRNAs that decode highly represented viral codons that are rare in host genes. While early stages of viral replication, including reverse transcription, transcription or integration are not significantly impacted, SLFN diminishes the synthesis of viral proteins, including Gag, Pol, Vif, Vpu and Vpr (46, 72). SLFN-mediated processes have downstream implications, including decreased viral particle production and blocking of viral latency reversal (44, 46, 73). SLFN11 and 12 endoribonuclease domains target type II tRNAs (tRNA^Ser^ and tRNA^Leu^) and tRNA^Leu^(UAA), respectively, as a DNA damage response in cancer cells (74, 75, 76). SLFN13 targets both tRNA and rRNA, restricting HIV replication (77).

### Changes in tRNA epitranscriptome upon viral infection

In addition to altered tRNA pools during viral infection, the tRNA epitranscriptome can also be reprogrammed (Fig. 1*B*) (Table 1). A global analysis of the RNA post-transcriptional modifications induced by positive-strand RNA viral infection showed significant changes in the overall cellular RNA modification landscape (78). Some of the modifications that were induced by viral infection were similar to stress-response-related changes. While the study did not specifically probe the tRNA modification landscape, some of the modifications detected in infected cells have previously been reported at the wobble position in tRNAs (Table 1) (79, 80). The fact that these modifications were not detected in the viral mRNAs suggests that the tRNA modification landscape is significantly altered upon viral infection, potentially influencing codon-anticodon interactions at the ribosome. DENV infection also induces temporal changes in specific AC loop modifications in the tRNA epitranscriptome of human Huh-7 cells (Table 1) (81).

**Table 1:** Modifications regulated upon viral infection or T-cell activation and their known or proposed functions

Modification^a^	tRNA^b^	Virus	Host cell	Source	Up/Down^c^	Method^d^	Function^e^	Ref
mcm^5^s^2^U	-	Poliovirus	Huh-7	Total RNA from virus-infected cells 5–7 hpi	Up	MS/MS	-	(78)
mnm^5^U	-	HCV	Huh-7	Total RNA from virus-infected cells 3 dpi	Up	MS/MS	-	(78)
ms^2^t^6^A mcm^5^s^2^U ncm^5^U	-	DENV	Huh-7	Total tRNA from virus-infected cells 1–32 hpi	Up	LC-MS/MS	-	(81)
f^5^Cm f^5^C	tRNA^Leu/CCA^	DENV ZIKV	Huh-7	Total tRNA from virus-infected cells	Down, 8 hpi; f^5^Cm up 24–32 hpi	LC-MS/MS	Translation recoding; promotes DENV replication	(81)
mcm^5^s^2^U ncm^5^U mcm^5^U	-	SARS-CoV-2	Vero6 Caco2	Total tRNA from virus-infected cells 24 hpi	Up	LC-MS/MS	-	(57)
I_m_ f^5^C mcm^5^U	-	SARS-CoV-2	Calu3	Total tRNA from virus-infected cells 32 hpi	Down, (I_m_, f^5^C); Up (mcm^5^U)	LC-MS/MS	Translation recoding; promotes viral replication	(60)
m^1^A14	tRNA^Phe/GAA^	SARS-CoV-2	Calu3	Total tRNA from virus-infected cells 32 hpi	Down	Mim-tRNA-seq	-	(60)
acp^3^U20	tRNA^Ile/AAU^	SARS-CoV-2	Calu3	Total tRNA from virus-infected cells 32 hpi	Down	Mim-tRNA-seq	-	(60)
m^1^G37	tRNA^Ala/UGC^	SARS-CoV-2	Calu3	Total tRNA from virus-infected cells 32 hpi	Down	Mim-tRNA-seq	-	(60)
t^6^A37	tRNA^Lys/UUU^	SARS-CoV-2	Calu3	Total tRNA from virus-infected cells 32 hpi	Down	Mim-tRNA-seq	-	(60)
I m^1^I ncm^5^U mcm^5^U t^6^A	-	HCoV-OC43	MRC5	Total tRNA from virus-infected cells 48 hpi	Down, (I, m^1^I, ncm^5^U, t^6^A); Up (mcm^5^U)	LC-MS/MS	Translation recoding; promotes viral replication	(60)
A_m_ m^1^A m^2^A m^2,2^G	-	HCoV-OC43	MRC5	Total tRNA from virus-infected cells 48 hpi	Down, (m^1^A, m^2^A); Up (A_m_, m^2,2^G)	LC-MS/MS	-	(60)
m^1^A14	tRNA^Phe/GAA^	HCoV-OC43	MRC5	Total tRNA from virus-infected cells 32 hpi	Down	Mim-tRNA-seq	-	(60)
t^6^A37	tRNA^Lys/UUU^	HCoV-OC43	MRC5	Total tRNA from virus-infected cells 32 hpi	Down	Mim-tRNA-seq	-	(60)
t^6^A ms^2^t^6^A m^1^A m^2,2^G m^6^t^6^A m^6^Am	-	SARS-CoV-2	ACE2-over-expressing HEK293	Total RNA from virus-infected cells 18 hpi	Up	LC-MS	-	(83)
t^6^A ms^2^t^6^A	-	SARS-CoV-2		COVID-19 patients' serum and urine	Up	LC-MS	-	(83)
m^1^A9	tRNA^Asp/GUC^ mito-tRNA^Val/UAC^	SARS-CoV-2		COVID-19 patients' nasopharyngeal swabs	Down	MSR	-	(85)
m^1^A58	tRNA^Glu/CUC^	SARS-CoV-2		COVID-19 patients' nasopharyngeal swabs	Down	MSR	-	(85)
m^1^A58	tRNA^Lys/UUU^	SARS-CoV-2		COVID-19 patients' nasopharyngeal swabs	Up	MSR	-	(85)
D20 D20 D19	tRNA^Val/CAC^ tRNA^Val/UAC^ tRNA^Gly/CCC^	HCMV	human foreskin fibroblasts	Small RNA from virus-infected cells 72 hpi	Down	tRNA-seq	-	(82)
ms^2^t^6^A37	tRNA^Lys/UUU^	HCMV	human foreskin fibroblasts	Small RNA from virus-infected cells 72 hpi	Down	tRNA-seq	-	(82)
m^3^C32	tRNA^Ser/UGA^	HCMV	human foreskin fibroblasts	Small RNA from virus-infected cells 72 hpi	Up	tRNA-seq	-	(82)
ms^2^t^6^A37 yW37	tRNA^L^^ys/UUU^ tRNA^Phe/GAA^	Anti-CD3/anti-CD28 activation	Mice CD4+ T cells	Small RNA	Down, upon activation; up, upon differentiation to memory cells	tRNA- seq LC-MS	maintaining PRF in HIV-1 Gag-Pol synthesis	(86)
m^1^A58	tRNA^Ser/GGA/GCU^ tRNA^Leu/CAA/CAG^	Anti-CD3 and anti-CD28 antibody activation	Mice CD4+ T cells	Small RNA	Up, upon activation	tRNA-seq LC-MS	enhance translation of proteins including MYC, guiding cells towards proliferative and expansion states	(89)
mcm^5^U	-	CHIKV	HEK 293T	Total tRNA from virus-infected cells 12 hpi	Up	LC-MS/MS	promotes viral RNA and protein synthesis	(55)
mcm^5^U	-	DENV	HEK 293T	Total tRNA from virus-infected cells	Up	LC-MS/MS	-	(55)
mcm^5^s^2^U R-mchm^5^U	-	ZIKV	Human primary astrocytes	Small RNA from virus-infected cells	Up, 48 hpi; down, 120 hpi	LC-MS/MS	promote viral protein synthesis and replication	(97)
mcm^5^s^2^U ncm^5^U S-mchm^5^U	-	ZIKV	SH-SY5Y	Small RNA from virus-infected cells	Up, 72 hpi; down, 96 hpi	LC-MS/MS	promote viral protein synthesis and replication	(97)
I i^6^A m^1^A ψ	-	Shewanella phage 1/4	*Shewanella glacialimarina*	Total tRNA from phage-infected cells	Down	UPLC-MS	-	(63)
t^6^A s^2^C	-	Shewanella phage 1/4	*Shewanella glacialimarina*	Total tRNA from phage-infected cells	Up, 30 min pi; down, afterwards	UPLC-MS	-	(63)
Q	-	Shewanella phage 1/4	*Shewanella glacialimarina*	Total tRNA from phage-infected cells	Down, 15 min pi; up, afterwards	UPLC-MS	viral MCP synthesis	(63)

a Modifications are defined as: 5-carbamoylmethyluridine, ncm^5^U; *N*6,2′-*O*-dimethyladenosine, m^6^Am; 2-thiocytidine, s^2^C; I_m_, 2′-*O*-methylinosine; m^1^I, 1-methylinosine; m^2^A, 2-methyladenosine; the rest of the modifications are defined as in the main text.

b A dash (−) indicates the tRNA source of the modification is not known.

c Regulation upon viral infection or T-cell activation.

d Methods are defined as: Tandem mass spectrometry, MS/MS; Multiplex small RNA sequencing, MSR; tRNA-sequencing, tRNA-seq; Ultra-performance LC-MS, UPLC-MS; Mim-tRNA-seq, modification-induced misincorporation tRNA-seq; the rest of the methods are defined as in the main text.

e A dash (−) indicates function unknown.

SARS-CoV-2 infection has also been shown to change the cellular tRNA landscape, although the results vary between studies (57, 60, 82). During SARS-CoV-2 infection in Vero6 and Calu3 cells, changes in certain modifications, including at the wobble position, were observed (Table 1) (57, 60). However, another report indicated no tRNA modification changes in the infection of Calu3 cells (82). These differences likely stem from variations in infection methods and efficiencies, as well as temporal differences in tRNA harvesting post infection.

In a study to identify modified nucleosides as potential biomarkers for SARS-CoV-2, levels of six RNA modifications were found to be elevated in infected HEK293 cells (Table 1) (83). The levels of *N6*-threonylcarbamoyladenosine (t^6^A) and methylthio-t^6^A (ms^2^t^6^A), which showed the most change, were also increased in the serum and urine of COVID-19 patients, correlating with symptom severity and duration. While the source of these modifications is unclear, t^6^A and ms^2^t^6^A are commonly found at position 37 in tRNAs (84).

Testing for their potential to be used as biomarkers for COVID-19 severity, the tRNA profile, including abundance, modification, and fragmentation was characterized from nasopharyngeal swab samples of individuals who tested positive for SARS-CoV-2 (85). While global tRNA abundance showed little variability among uninfected vs SARS-CoV-2-infected patients, several isodecoders were reduced in infected samples. The study also reported variability in 1-methyladenosine (m^1^A) modification levels at different tRNA isoacceptor positions in healthy vs symptomatic individuals (Table 1). As this study explored the potential of using tRNAs as a diagnostic tool for SARS-CoV-2, the focus was on secreted, extracellular tRNAs. Another limitation is that this study only focused on modifications producing a reverse transcription signature. Nevertheless, these studies suggest that the tRNAome is impacted during SARS-CoV-2 infection, likely in a tissue-tropic manner (57, 82, 83, 85). Aharon-Hefetz *et al*. characterized the temporal changes in the tRNAome upon human cytomegalovirus (HCMV) infection in human foreskin fibroblast cells (Table 1) and parsed out virus-induced changes from host immune responses in the tRNA epitranscriptome (82). The virus was identified as the main contributor to changes in the tRNA pool. Dihydrouridine (D) levels in the D loop and AC stemloop modifications showed changes upon HCMV infection.

Changes in the tRNA pool, modifications, and codon usage were reported during T-cell activation in mice (86). Following antigen receptor triggering in the early stage of proliferation, both codon usage and tRNA expression levels changed in a manner that promoted cell proliferation. Upon differentiation, tRNAs returned to the basal level, potentially to restrict excessive proliferation. Using abortive or mismatch reverse transcription signatures, five out of the 18 annotated mice tRNA modifications in the MODOMICS database could be detected and quantified during the T-cell activation process. While most modifications remained unchanged, ms^2^t^6^A and wybutosine (yW) levels at position 37 in the AC loop of tRNA^Lys^(UUU) and tRNA^Phe^(GAA), respectively, decreased in activated T-cells and increased again upon differentiation to memory cells. tRNA^Arg^(UCU) isodecoders also showed changes in 3-methylcytidine (m^3^C) levels at position 32. The altered modifications at position 37 may influence reading frame maintenance and decoding slippery codons, as discussed in more detail later.

### Changes in tRNA epitranscriptome upon viral infection impact translation

The reprogramming of the cellular tRNA pool during infection leads to multiple downstream effects, including translation upregulation of stress response proteins and viral proteins (Fig. 1*B*) (54). These effects are mainly mediated through modifications at the wobble position, which restrict/expand the decoding capacity, influence protein homeostasis and/or translation kinetics (6, 12, 87). Some viral genes have similar codon usage as host cell cycle regulation and stress response genes, suggesting a viral adaptation for efficient translation by exploiting host responses to infection (55, 88).

How a specific tRNA modification drives T-cell proliferation through signal-dependent, codon-specific reprogramming of protein synthesis has been explored (89). During the early stages of naïve T-cell transitioning to hyperactive states, levels of tRNA m^1^A58 writer proteins, tRNA methyltransferase 61A and 6 (TRMT61A and TRMT6) are upregulated, causing the modification to be installed on a subset of tRNAs (Table 1). These tRNAs are preferentially used to improve the translation efficiency of proteins that promote rapid metabolic reprogramming and T-cell expansion, including the myelocytomatosis (MYC) protein.

Reprogramming of the tRNA epitranscriptome to match viral codon usage was also investigated in the positive-strand RNA virus, CHIKV (8, 55). CHIKV genes show an A/U-ending codon usage, differing from the G/C-ending codon bias in highly expressed human mRNAs. Upon viral infection of HEK 293T cells, certain cellular mRNAs were translationally downregulated, while mRNAs related to cell cycle, RNA transport and DNA damage response were activated. The codon usage (GAA (Glu), AAA (Lys), CAA (Gln) and AGA (Arg)) of these activated host genes and CHIKV genes are similar, while repressed genes have a more typical host codon usage, suggesting viral-induced codon reprogramming. These enriched codons are decoded by tRNAs containing 5-methoxycarbonylmethyluridine (mcm^5^U) and 5-methoxycarbonylmethyl-2-thiouridine (mcm^5^s^2^U) modifications at position 34. GGA (Gly), another enriched codon, is decoded by tRNAs containing mcm^5^U and 5-methoxy-carbonyl-hydroxy-methyluridine (mchm^5^U) modifications at the wobble position (15, 87, 90, 91, 92). Interestingly, one of the most translationally-activated host genes is *TRM9b/TRM9L/KIAA1456*, a predicted mammalian homolog of the *Saccharomyces cerevisiae* Trm9 protein, which converts cm^5^-modified U34 to mcm^5^U34. KIAA1456 has been identified as a tumor growth suppressor and a regulator of synaptic development and neurotransmission (93, 94, 95). While ALKB homolog 8 (ALKBH8) is established as the main mcm^5^U-modifying enzyme, and the overall mcm^5^U modification levels were not affected upon KIAA1456 deletion in colon carcinoma cells and *drosophila* (23, 93, 95), deletion of the KIAA1456 methyltransferase domain disrupted its synaptic growth function (93). This suggests that under certain conditions, this protein may be involved in the modification of a subset of tRNAs. During CHIKV infection, upregulation of mcm^5^ at U34 due to KIAA1456 overexpression supported increased viral RNA and protein levels. As the enzyme itself is enriched in the optimally expressed codons, protein expression engages in a positive feedback loop. In yeast, alkylation stress induces expression of DNA damage and cell cycle control genes enriched in GAA and AGA codons through upregulation of tRNA modifications in a Trm9-dependent pathway (96). Enrichment of the same codons and genes with similar functions upon CHIKV infection may suggest a link between the evolution of codon optimization of the virus and host stress responses.

The DENV genome shows a similar codon enrichment as CHIKV, and viral infection led to higher expression of KIAA1456 and mcm^5^U modification levels in HEK 293T cells, suggesting a similar codon-reprogramming event linked to tRNA modification change (55). Chan *et al*. identified host ALKB homolog 1 (ALKBH1), which is involved in 5-formylcytidine (f^5^C) and 5-formyl-2′-*O*-methylcytidine (f^5^Cm) synthesis at position 34 in tRNA, as a DENV restriction factor in Huh-7 cells. Modulation of ALKBH1 protein levels was reported to affect viral protein synthesis through UUA (Leu) codon recoding (81). DENV NS5 protein was involved in 2′-*O* methylation of f^5^C34 in tRNA^Leu^(CAA) in late-stage viral infection (Table 1) (81).

A similar mechanism has been proposed in SARS-CoV-2, where the genome is enriched in A/U-ending codons, and wobble modifications are regulated to promote viral protein synthesis (Table 1) (57, 60). In a similar mechanism, the Zika viral genome encodes an AA-ending codon bias, deviating from the host's AG-ending bias; viral infection increased mcm^5^s^2^U34 modification in host tRNAs, supporting viral protein synthesis and replication (97, 98).

Temporal characterization of the tRNA modification landscape of the cold-active marine bacterium, *Shewanella glacialimarina*, upon Shewanella phage 1/4 infection revealed modification dynamics, which may correlate with the differential codon usage of early and late stage phage gene expression (63). Changes in AC loop modifications such as inosine (I) at position 34, and isopentenyl adenosine (i^6^A) and t^6^A at position 37 may impact viral and host protein synthesis. Notably, queuosine (Q) modification found at position 34 of tRNA(GUN) increased as viral infection progressed. Since late phage genes, such as the gene encoding the major capsid protein (MCP), show a bias for the UAC codon decoded by Q34-modified tRNA^Tyr^(GUA), elevated Q levels may promote late-stage phage infection by facilitating efficient MCP synthesis.

## tRNA modifications in frameshifting events in viruses

A subset of retroviruses, positive-strand RNA viruses, double-stranded (ds) RNA viruses infecting yeast, plant RNA viruses, retrotransposons, and bacteriophages employ PRF, where the reading frame is shifted at specific sequences to produce multiple proteins from the same mRNA. For compact viral genomes, this allows modulation of viral gene expression in the host cell (99, 100, 101, 102, 103). Most retroviruses and retrotransposons contain overlapping Gag and Gag-Pol polyprotein sequences, where Gag and Pol regions encode for viral structural and enzymatic proteins, respectively. Gag-Pol is synthesized either *via* readthrough suppression of a termination codon or ribosomal frameshifting in the −1 or +1 direction (102, 104). Gag and Gag-Pol are generated at a 20:1 ratio; this ratio is conserved among retroviruses, which allows for increased expression of the more abundantly required major structural proteins relative to the viral enzymes encoded by *pol* (105). The fusion protein also provides a clever mechanism to ensure incorporation of the enzymes into viral particles (104). Maintaining the Gag:Gag-Pol ratio as well as efficient frameshifting is critical for HIV-1 infectivity and replication, indicating its potential as a therapeutic target (105, 106).

In −1 frameshifting events observed in mouse mammary tumor virus (MMTV), Rous sarcoma virus, HIV-1 and HTLV-1, the frameshifting site is marked by a heptanucleotide “slippery” sequence containing two homopolymeric triplets (XXXYYYZ, where X can be any nt, Y is A/U and Z is anything but G) and either a pseudoknot (more common) or a stem-loop structure 6 to 10 nt downstream of the slippery sequence (102, 104). −1 ribosomal frameshifting occurs upon simultaneous slippage of two adjacent ribosome-bound tRNAs from the 0-reading frame (X_XXY_YYZ) to the −1 frame (XXX_YYY_Z). After the −1 shift, tRNAs remain base-paired to the mRNA in at least two of the three anticodon positions, and once the peptidyl-tRNA is transferred from the A to the P ribosomal site, translation continues in the −1 frame. The downstream pseudoknot or stem-loop structures slow down the translating ribosomes and facilitate frameshifting by causing pausing at the slippery site (100, 102, 104, 107, 108).

The 3′ terminal codons commonly found at the −1-frameshifting site in retroviruses are AAC, UUA and UUU. In addition to these codons, AAA, AAU and UUC are also found in eukaryotic frameshifting systems (104). tRNA AC modifications are well-established to regulate ribosomal frameshifting events (15). Except for the tRNA^Leu^ isoaccceptor that decodes UUA, other tRNAs that decode these codons are modified at either position 34 or 37—nt 34 in tRNA^Asn^ (decoding AAC and AAU) is modified to Q, nt 37 in tRNA^Phe^ (decoding UUU and UUC) is modified to yW, and nt 34 in tRNA^Lys^ (decoding AAA) is modified to mcm^5^s^2^U (Table 2) (104, 109). These tRNA AC loop modifications have been implicated in maintaining the reading frame and regulating frameshifting (110).

**Table 2:** Effect of tRNA modifications on viral programmed ribosomal frameshifting

Modification^a^	tRNA	Codon	Virus	Effect on viral replication	Ref
Q34	tRNA^Asn^	AAC AAU	Infectious bronchitis virus	Hypomodification had little effect on frameshifting	(102, 114)
Q34	tRNA^Asn^	AAC AAU	HTLV-1 BLV	Q levels decreased in virus-infected H9 cells	(109)
yW37	tRNA^Phe^	UUU UUC	*S*. *cerevisiae* L-A virus	Hypomodification increased frameshifting	(115)
yW37	tRNA^Phe^	UUU UUC	HIV-1	tRNA^Phe^ yW levels were lower in virus-infected H9 cells; PRF is higher when tRNA is hypomodified	(86, 109)
m^1^G37	tRNA^Phe^	UUU UUC	MMTV	Promotes −1 frameshifting when substituted for yW37	(121)
s^2^U34	tRNA^Lys^	AAA	Lambda phage	Levels modulate phage protein synthesis and phage production; hypomodification protects from phage infection	(122, 123)

a Modifications are defined as in the main text.

While Q34 in tRNA^Asn^ (decoding AAC/AAU codons), generally downregulates frameshifting, its effects are impacted by tRNAs occupying adjacent codons and their modifications, as well as its source (yeast *vs* mammalian) (111, 112). Frameshifting efficiency at AAC or AAU codons did not change when Q34-containing tRNA^Asn^ was microinjected into *Xenopus* oocytes (113). Similarly, when the slippery sequence variants of the frameshift signal of infectious bronchitis virus were expressed in *Escherichia coli* or horse serum, hypomodified Q34 in tRNA^Asn^ had little effect on frameshifting (101, 114). However, Q levels were reported to be decreased in tRNA^Asn^ in HTLV-1- and bovine leukemia virus (BLV)-infected H9 cells, which contain a 3′ AAC codon in their Gag-Pro and Pro-Pol frameshift signals, indicating tRNA modification levels may be adjusted to support frameshifting (Table 2) (109). Further studies in relevant host systems are needed to confirm the role of Q34 in viral PRF.

The yW37 modification in tRNA^Phe^ suppresses frameshifting events, particularly at the frameshift site codon, although the effects are again modulated by adjacent codons and other tRNA modifications (111). In a −1 frameshifting slippery-site reporter derived from the dsRNA *S*. *cerevisiae* L-A virus, hypomodification of yW37 in tRNA^Phe^ increased frameshifting efficiency, with progressive changes in frameshifting observed with each intermediate in the yW modification pathway (Table 2) (115). This result is consistent with a stepwise evolution and tuning of frameshift potential. A similar result was observed in *Xenopus* oocytes, where microinjection of tRNA^Phe^ containing yW inhibited frameshifting (113). −1 ribosomal frameshifting was increased in human colorectal cancers due to loss of tRNA-yW synthesizing protein 2 (TYW2) that incorporates yW in tRNA^Phe^ (116).

As noted above, the level of yW at position 37 in tRNA^Phe^(GAA) is reduced during T-cell activation (86). In a HeLa cell line with reduced yW modification due to deletion of a yW writer, ribosomal frameshifting at UUU (−1) was increased in a HIV Gag-Pol frameshifting reporter (86). While the T-cells may hypomodify the tRNAs as a tradeoff between translation fidelity and speed in rapidly proliferating cells, HIV-1 viruses infecting proliferating T-cells benefit from the hypomodification as it allows for more efficient −1 PRF necessary for Gag-Pol synthesis (86). Chromatographic analysis revealed that yW levels were indeed lower in tRNA^Phe^ from HIV-1-infected H9 cells (Table 2) (109). tRNA^Leu^ that decodes the UUA codon in the HIV-1 PRF sequence is also rare in human T cells; this fact, coupled with the presence of hypomodified tRNA^Phe^, supports HIV-1 frameshifting and ensures the proper frameshift frequency (117).

The 1-methylguanosine (m^1^G) modification at position 37 of all tRNAs that read codons starting with C, except those that read CAN codons, downregulates frameshifting in *Salmonella typhimurium* in a mechanism common to eukaryotes and archaebacteria (118, 119). Recent structural work showed how m^1^G37 in *E*. *coli* tRNA^Pro^ suppresses +1 ribosomal frameshifting (119, 120). Additionally, m^1^G in place of yW at position 37 in tRNA^Phe^ promoted −1 frameshifting in the MMTV Gag-Pro frameshift signal in an *in vitro* reporter system, while the overall modification environment in the AC loop impacted the degree of frameshifting (Table 2) (121).

A study of lambda phage infection of *E*. *coli* shed light on a complex network linking sulfur metabolism, tRNA modification, and PRF that promotes viral susceptibility. The iron-sulfur (Fe-S) cluster biosynthesis pathway exerts a protective effect on *E*. *coli* during phage infection, while a tRNA thiolation pathway enhances viral infection. tRNA^Lys^(UUU) 2-thiouridine (s^2^U34) modification, modulated by sulfur uptake, regulates PRF to maintain the optimal ratio of phage proteins gpG and gpGT, while hypomodification of U34 led to increased frameshifting, disrupting the phage protein ratio and viral production (Table 2) (122, 123).

Taken together, these results suggest that tRNA modifications in the AC loop are generally unfavorable for frameshifting. Therefore, to promote PRF events that are essential for viral protein synthesis and efficient replication, viruses may influence the modification landscape in the AC region of tRNAs. These effects are expected to be specific to the tRNAs that engage with codons at slippery sequences in viral PRF-sites, as overall hypomodification effects could impact global translation fidelity and efficiency.

## Modifications in tRNAs packaged into virus particles

In addition to viral genomic RNA, many viruses package specific host RNAs. In retroviruses, non-coding RNAs (ncRNAs) such as 7SL RNA, 5S RNA, spliceosomal U6 small nuclear RNA (snRNA) and tRNAs are all found in virions, while tRNAs and signal recognition particle (SRP) RNA have been detected in SARS-CoV-2 viral particles (124, 125). However, the function of ncRNAs, including non-primer tRNAs, their mechanism of selective packaging and the status and potential function(s) of modifications remain mostly unexplored. A deep-sequencing study estimated that tRNAs constitute more than 96% of host RNAs in HIV-1 viral particles (126). Besides primer tRNA^Lys,3^(UUU) and tRNA^Lys,1,2^(CUU), tRNA^Asn^(GUU), tRNA^Asp^(GUC), and tRNA^Ile^(UAU), which decodes a rare codon in cellular mRNAs, are reported to be enriched in HIV-1 virus particles (53, 124, 126, 127).

**Figure 2:**
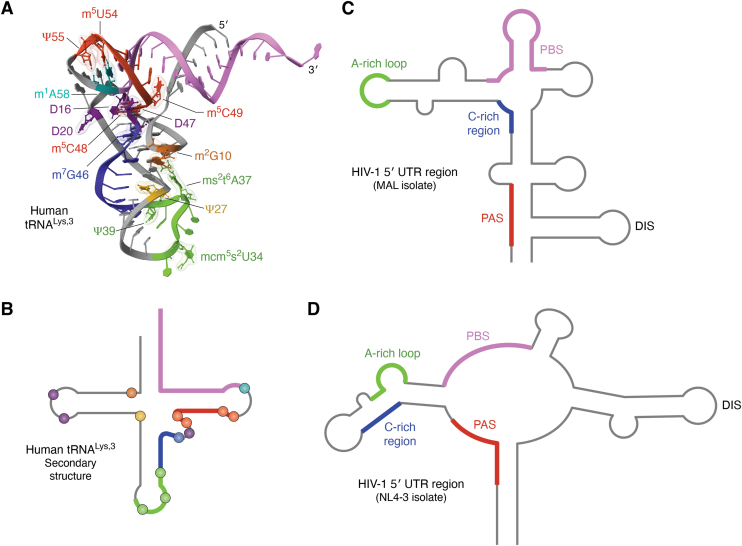
**Interactions of tRNA^Lys,3^ with HIV-1 5′ UTR during reverse transcription initiation**. *A*, tertiary structure of human tRNA^Lys,3^ (taken from the cryo-EM structure of human LysRS bound to cellular modified tRNA^Lys,3^ and AIMP2 peptide complex, PDB code: 9DPL) with known modifications. The sites complementary to or known to interact with the HIV-1 5′ UTR are colored: 3′ 18 nt of tRNA^Lys,3^ (*pink*), part of TψC stem (*red*), part of AC stemloop (*green*) and variable loop and part of AC stem (*blue*). Sites of known modifications are indicated by colored ball and stick representations. *B*, secondary structure of human tRNA^Lys,3^. The sites complementary to or known to interact with the HIV-1 5′ UTR are colored as in Figure 2*A*. Modification sites are indicated as circles and colored as in Figure 2*A*. *C*, secondary structure model of the 5′ UTR region of HIV-1 MAL isolate. The sites complementary to or known to interact with the tRNA^Lys,3^ primer are colored corresponding to Figure 2*A*: PBS (*pink*), A-rich loop (*green*), C-rich region (*blue*) and PAS (*red*). The dimerization initiation site (DIS) is also indicated. *D*, secondary structure model of the 5′ UTR region of HIV-1 NL4-3 isolate. The sites complementary to or known to interact with the tRNA^Lys,3^ primer are colored as in *panel B* and the DIS is also indicated.

All human tRNA^Lys^ isoacceptors are selectively packaged into HIV-1 virions through interactions between HIV-1 Gag and the cognate lysyl-tRNA synthetase (LysRS) (128). A recent cryo-EM structure of fully modified human tRNA^Lys,3^ bound to human LysRS revealed the presence of all 14 previously identified modifications and their role in formation of the complex (Fig. 2*A*) (129). Binding studies showed that the presence of the modifications enhances the affinity for LysRS by 2.5-fold (129). The tRNA^Lys,3^ AC-loop is extensively remodeled relative to the unmodified transcript, and loop modifications mcm^5^s^2^U34 and ms^2^t^6^A37 play an integral role in recognition by LysRS. Based on the structure of the complex, the role of highly conserved D modification, found in tRNAs from all domains of life, was revealed for the first time. In the unmodified tRNA, U20 in the D-loop and U47 in the variable loop stack with each other in the tRNA central core. Modification to D leads to a loss of the planar nature of these pyrimidine bases and in their ability to stack. The unstacked conformation creates a wider groove between the D-loop and the variable loop and allows the N-terminal domain (NTD) of LysRS to dock into the newly formed groove. The structured NTD helix was observed for the first time and only in the complex with fully modified tRNA. The role of modifications in tRNA primer packaging into HIV-1 particles remains to be explored.

Early work in avian retroviruses investigated how modifications in specific tRNA primers impact their packaging into virions. *N2*-methylguanosine (m^2^G) at position seven in the acceptor stem of primer tRNA^Trp^ was reported to inhibit packaging (130). This suggested a mechanism where factors involved in selective packaging of the tRNA primer are unable to recognize the modified tRNA or are actively excluding the modified tRNA from being packaged.

Host cytosolic pattern recognition receptors such as toll-like receptors (TLRs) in human cells sense unmodified RNA as foreign and trigger innate immune responses against them (131). Only one in 50 virions in HIV-1 infected cells are estimated to lead to productive infection and capsid (CA) stability mutants show significant defects in nuclear import of pre-integration complexes (132). More recent worked showed that infectious viral cores enter the nucleus largely intact and complete reverse transcription in the nucleus prior to uncoating near integration sites (133, 134). However, most viral particles fail to complete these steps. A double-labeling strategy that enabled the visualization of uncoating of single cores in living cells showed that uncoating occurs in the cytoplasm at a faster rate than nuclear uncoating, suggesting that the CA lattice is stabilized by nuclear factors (135). Thus, packaging of modified tRNAs may benefit viruses by helping to evade immune responses upon cytoplasmic uncoating; the presence of 5-methylcytidine (m^5^C), *N6*-methyladenosine (m^6^A), 5-methyluridine (m^5^U), s^2^U and pseudouridine (ψ) on packaged tRNA may help to perceive the RNA as self (125, 136). Indeed, it has been shown that 1,2′-*O*-dimethylguanosine (m^1^Gm) at position 18 of *E*. *coli* tRNA^Tyr^ (137, 138), as well as 5,2′-*O*-dimethyluridine (m^5^Um) at position 54 of mammalian tRNA^Lys,3^ suppresses TLR7-activated immunostimulation in human peripheral blood mononuclear cells (139).

A comprehensive characterization of the modification profile of packaged tRNAs in retroviral virions is currently lacking. A liquid chromatography-mass spectrometry (LC/MS) analysis of RNA from HIV-1 virions coupled with m^1^A profiling and deep sequencing revealed that m^1^A modification is found on packaged tRNAs (126). In addition, t^6^A, 2′-*O*-methyladenosine (Am) and ms^2^t^6^A, which are commonly present in tRNAs, were detected (126). Whether m^1^A and/or other modifications on non-primer tRNAs in the virus serve any function warrants further investigation.

Modified 5′ and 3′ fragments of specific tRNAs were detected in *Iflaviridae* family viruses that infect honeybees (140). m^1^A was detected at positions 59 and 56 in tRNA^Lys^(CUU) and tRNA^Gly^(GCC) fragments respectively, while m^5^C was detected at 100% occupancy at specific positions in tRNA^Asp^(GUC), tRNA^Lys^(UUU) and tRNA^Glu^(CUC) fragments. The biological implications of the presence of modified tDRs in honeybee-infecting virus particles are not understood.

The identity and modification levels of tRNAs packaged into SARS-CoV-2 viruses from cultured Vero cells showed that specific tRNA isoacceptors are selectively enriched in virion particles (125). Compellingly, the modification patterns were different for packaged tRNAs *versus* their cellular counterparts. While m^1^A58 levels were higher in packaged tRNA^Leu^(AAG) and tRNA^Lys^(UUU), packaged tRNA^Glu^(UUC) isodecoders showed low levels of m^1^A58. tRNA^Leu^(AAG) and tRNA^Ser^ (AGA) are more than 90% modified with I34 in both cells and virions. Packaged tRNA^Ser^ showed lower modification levels in the AC region, including *N6*-methyl-*N6*-threonylcarbamoyladenosine (m^6^t^6^A37). These results suggest selectivity for modification levels in packaged tRNAs, likely at the packaging step.

A recent structural study elucidated a key chaperone role for human tRNA^Gln^ and tRNA^Arg^ (referred to as tRNA^Gln/Arg^) with a specific modification profile in poxviral transcription-processing complex assembly (141). tRNA^Gln/Arg^ chaperone the assembly of the complete viral RNA polymerase transcription complex that includes transcription factors and mRNA processing proteins. These tRNAs also facilitate subsequent packaging of the complex into virions through a conformational shift in the AC-stemloop (141, 142). While cryo-EM densities matching certain tRNA modifications, such as m^1^A57 and 2′-*O*-methylcytidine (C_m_) at position 32, were identified in the complex, mcm^5^s^2^U34, a prevalent modification in tRNA^Gln^ was lacking. The presence of this modification would result in unfavorable interactions with interacting proteins. Interestingly, LC-MS/MS revealed global upregulation of the wobble base modification upon viral infection, suggesting that selective mechanisms are at play to ensure that tRNAs with the desired modification pattern are recruited to form the complex (141).

## Role of modifications in tRNA primers during retroviral reverse transcription initiation

Retroviruses use a specific host tRNA to serve as the primer for reverse transcription initiation (143). The 3′ 18 nt of the tRNA primer anneals to a complementary primer binding site (PBS) sequence in the 5′ UTR of the viral RNA in a reaction mediated by viral and/or host chaperone proteins (144). In HIV-1, the Gag protein mediates primer tRNA^Lys,3^ unwinding and annealing, primarily through the nucleocapsid (NC) domain (145, 146, 147). Reverse transcriptase (RT) binds to the newly formed ds primer-template complex and extends from the 3′ hydroxyl of the terminal A76 of the tRNA during the initiation step of cDNA synthesis (144). In HIV-1, additional regions of tRNA-viral RNA complementarity that involve modified nt in the tRNA have been reported (Fig. 2) (148, 149, 150, 151, 152). Although these secondary primer–template interactions are known to influence the initiation step in different retroviruses, the role of tRNA modifications in this process is not well understood (153, 154).

In the HIV-1 MAL isolate, a specific interaction between an A-rich loop upstream of the PBS and the AC loop of primer tRNA^Lys,3^ has been proposed to facilitate effective reverse transcription initiation and viral infectivity (Fig. 2, *A* and *B*, *C*) (155, 156). Attempts to switch HIV-1 primer specificity by mutating the PBS sequence were not successful, as the virus reverted to encode a PBS complementary to the 3′ end of tRNA^Lys,3^. Stable viruses that use alternative primers such as tRNA^His^ could be obtained by substituting both the PBS sequence and the A-rich loop sequence to match the corresponding tRNA’s 3′ 18 nt sequence and AC sequence, respectively (157). This result supports A-rich loop-AC interactions.

An *in vitro* RNA secondary-structure probing study of the 5′ UTR of HIV-1 MAL annealed to native, fully modified, bovine liver tRNA^Lys,3^ also supported a specific interaction between the A-rich loop and the tRNA^Lys,3^ AC loop containing the ^33^Umcm^5^s^2^UUU^36^ sequence (Fig. 2, *A* and *B*, C) (154). This interaction was absent or destabilized when an *in vitro* transcribed tRNA was used or mcm^5^s^2^U was dethiolated, respectively. A surface plasmon resonance study showed that mcm^5^s^2^U34 and ms^2^t^6^A37 strongly stabilized the interaction between the A-rich loop and the tRNA^Lys,3^ AC-stemloop (158). Recombinant human tRNA^Lys,3^ expressed in *E*. *coli* containing a s^2^U34 modification was more efficient in reverse transcription initiation compared to a 5-methylaminomethyluridine (mnm^5^U34)-containing tRNA. This is consistent with the role of thiolation at U34 in initiation (159). Rak *et al*. reported that the ms^2^t^6^A modification level at position 37 in tRNA^Lys^(UUU) is decreased during T-cell activation (86). However, whether the modification level is upregulated upon HIV-1 infection is unknown. Modifications on tRNA^Lys,3^ are also implicated in efficient transition from the initiation to elongation step of reverse transcription (153).

HIV-1 NC protein was reported to bind with ∼10-fold higher affinity to human tRNA^Lys,3^ containing mcm^5^s^2^U34 and ms^2^t^6^A37, compared to unmodified tRNA (160). NC denatured the structure of the modified tRNA more effectively than unmodified tRNA, suggesting that the AC modifications may facilitate NC-mediated primer annealing to the HIV-1 genome (160). The development of peptides with enhanced binding activity to the modified tRNA^Lys,3^ AC loop, thus competing for NC binding and inhibiting primer annealing to the viral genome, has been explored (161).

In HIV-1 subtype B viral genomes such as HXB2 and NL4-3, an alternate to the A-rich loop-AC interaction has been proposed involving the TψC arm of tRNA^Lys,3^ and a sequence upstream of the PBS called the primer activation signal (PAS) sequence (Fig. 2, *A* and *B*, *D*) (162, 163). While the TψC arm of tRNA^Lys,3^ contains ψ at position 55, m^5^Um at position 54, and m^5^C at positions 48 and 49, the effect of these modifications on this interaction is unclear (162, 164). A cryo-EM structure of the HIV-1 RT initiation complex using unmodified tRNA^Lys,3^ annealed to an ∼100-nt PBS-containing region of the HIV-1 NL4-3 RNA, revealed that the tRNA folds into a noncanonical, extended helical form (151). This structure shows extended base pairing interactions beyond the 18-nt PBS that involve A58, which is modified to m^1^A58 in native tRNA^Lys,3^. The methyl group at *N1* of m^1^A projects from the Watson-Crick face of adenosine and prevents base-pairing interactions (165). How the presence of this modification influences the structure of the initiation complex has not been tested.

Additional interactions involving modified nt in the tRNA primer and the viral RNA have also been reported in other retroviruses. The 5′ UTR U5 region RNA of avian sarcoma and leukosis virus (ASLV) is proposed to form a base-pairing interaction with the TψC stemloop of an *in vitro* transcribed tRNA^Trp^ primer (150). However, the impact of two consecutive ψ sites in the TψC stemloop as well as adjacent modifications in the native tRNA primer on the base-pairing interaction is unknown. In Moloney murine leukemia virus (M-MuLV) additional primer-template interactions outside of PBS were observed when an *in vitro* transcribed tRNA^Pro^ primer was annealed. These interactions were absent in the fully modified, native primer-template complex, indicating that in this system the modifications prevent additional contacts outside of the PBS (166).

## Role of modifications in tRNA primers during retroviral strand-transfer

**Figure 3:**
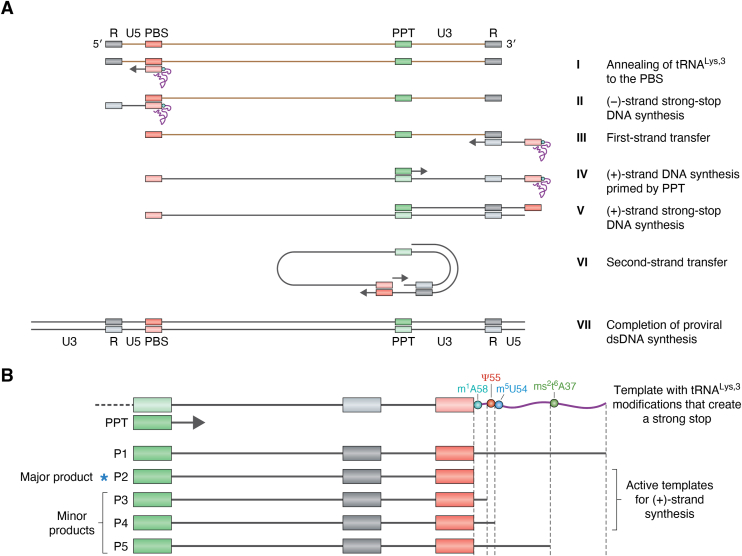
**Role of tRNA modifications in retroviral reverse transcription**. *A*, scheme of HIV-1 reverse transcription. The repeated sequence (R), 5′ unique sequence (U5), primer binding site (PBS), polypurine tract (PPT), and 3′ unique sequence (U3) are labeled. Viral RNA sequence is in *brown* and DNA sequences are in *black*. Following DNA synthesis, RNA strands in RNA-DNA duplexes are hydrolyzed by RNaseH activity of RT. I: Annealing of 3′ 18 nt of tRNA^Lys,3^ to the PBS. II: Minus-strand strong-stop DNA synthesis at the 5′ end of the viral genome. III: First-strand transfer. IV: Plus-strand DNA synthesis primed by the PPT. V: Plus-strand strong-stop DNA synthesis. VI: Second-strand transfer. VII: Completion of double-stranded proviral DNA synthesis. *B*, the strong stop in plus-strand DNA synthesis (Step V in panel 3A) is modulated by tRNA^Lys,3^ modifications. The template for plus-strand DNA synthesis is shown with the tRNA^Lys,3^ modifications known to generate a strong stop: m^1^A58, Ψ55, m^5^U54 and ms^2^t^6^A37, primed by the PPT sequence (*green*). Five strong-stop products (P1-5) are shown based on the modifications in the template: P1—when the tRNA is unmodified, reverse transcription proceeds to the 5′ end of the tRNA; P2—plus-strand synthesis terminates before m^1^A58; P3—plus-strand terminates at Ψ55; P4—plus-strand terminates at m^5^U54; P5—plus-strand terminates at ms^2^t^6^A37. P2 is the major product in HIV-1 reverse transcription. P3-5 are minor products. Only P2-4 can serve as active templates.

During retroviral reverse transcription, the minus-strand cDNA is generated after the highly modified tRNA^Lys^ primer (Fig. 2*A*) is annealed to the genomic RNA PBS (Fig. 3*A*, step I) and extended to generate a minus-strand strong-stop product (Fig. 3*A*, step II). This is followed by a first strand-transfer event and completion of minus-strand synthesis (Fig. 3*A*, step III). The RNA of the RNA-DNA hybrids generated during this process is degraded by the RNaseH activity of RT. The minus-strand serves as the template for plus-strand cDNA synthesis, primed by an RNaseH-resistant sequence called the polypurine tract (PPT) (Fig. 3*A*, step IV) (167, 168). The initial plus-strand synthesis step terminates at the end of the 3′ 18-nt tRNA primer sequence in the template due to the presence of tRNA modifications, producing plus-strand strong-stop (+sss) DNA (Fig. 3*A*, step V). This ensures that the 3′ end of the plus-strand has perfect complementarity to the PBS sequence at the 5′ end of the minus strand. Through a second strand-transfer event, these complementary regions base pair with each other and the plus strand serves as the primer for the completion of minus-strand cDNA synthesis, generating the complete cDNA of the retroviral genome (Fig. 3*A*, step VI and VII) (168, 169).

In HIV-1, the precise + sss cDNA synthesis product prior to the second strand-transfer step has been attributed to the presence of m^1^A modification at position 58 in tRNA^Lys,3^ (Fig. 3*B*, P2) (169, 170). In the native tRNA structure, m^1^A58 forms a reverse Hoogsteen base pair with m^5^Um54 in the TψC loop (171). A conserved modification across bacterial, archaeal and eukaryotic tRNAs, m^1^A58 is implicated in maintaining tRNA stability, as observed for initiator tRNA^Met^ in yeast (172, 173). This modification is present in all primer tRNAs (tRNA^Pro^, tRNA^Trp^, tRNA^Lys,3^, tRNA^Gln^, tRNA^His^, tRNA^Asn^, tRNA^Arg^, and tRNA^Ser^) used by retroviruses and some long-terminal repeat transposons, indicating a conserved function in retroviral reverse transcription (104).

*In vitro* studies by multiple groups using native, fully modified tRNA^Lys,3^ showed that the major product of HIV-1 +sss DNA synthesis terminated at m^1^A58, while *in vitro* transcribed tRNA was fully copied (Fig. 3*B*, P1) (170, 174, 175, 176, 177). Additional minor products corresponding to termination at ms^2^t^6^A37 (Fig. 3*B*, P5) (170) in the AC loop and ψ55 (Fig. 3*B*, P3) and m^5^Um54 (Fig. 3*B*, P4) in the TψC loop have also been identified (170, 174, 176). While the bulkiness of the modification moiety likely halts DNA synthesis at ms^2^t^6^A37, the cause of termination at ψ55, which can form Watson-Crick base pairs, is unclear (176). Mutant A58U tRNA^Lys,3^ significantly inhibited replication kinetics of HIV-1 in human T lymphoblast CEM cells, supporting the role of modified A58 in generating a viable strong-stop cDNA product *in vivo* (178). A more recent study in HEK293T cells showed that when the TRMT6-TRMT61A methyltransferase complex that incorporates m^1^A58 is mutated, plus-strand synthesis extended beyond A58 (177), confirming the role of tRNA^Lys,3^ m^1^A58 in +sss cDNA synthesis during HIV-1 reverse transcription. These TRMT6 mutant cells also had lower HIV-1 infectivity (177). Only the minus-strand products terminated at m^1^A58, m^5^Um54 and ψ55 could serve as functional templates for the subsequent plus-strand synthesis. Incomplete m^1^A modification at A58 leads to increased DNA synthesis to ψ55. These species still result in effective plus-strand transfer as lentiviruses including many HIV strains and spumaviruses contain a consensus, complementary sequence immediately adjacent and downstream to the PBS termed the primer over-extension sequence (174, 176, 177). This sequence illustrates the co-evolution of viral genomes to match the host tRNA primers' modification landscape. Modification circuitry in the TΨC loop involving m^1^A58, m^5^Um54 and Ψ55 likely played a role in the co-evolution process (177, 179).

## Modulation of tRNA pool and modifications during HIV-1 replication

**Figure 4:**
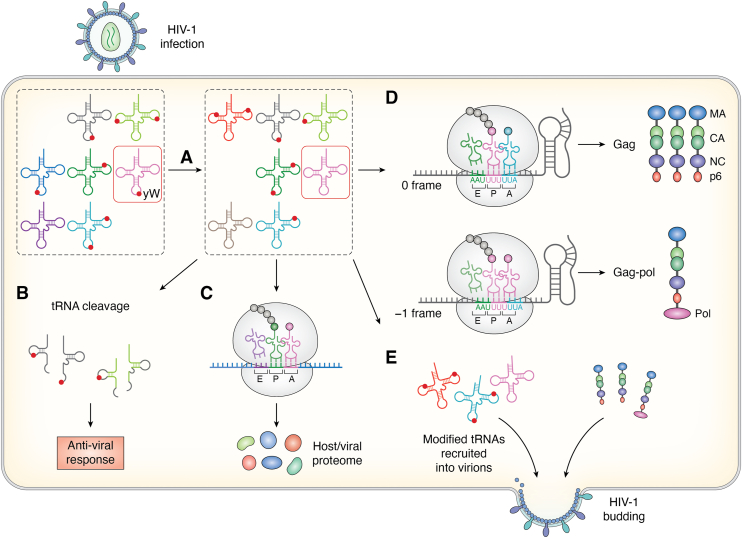
**Known and potential functions and regulation of tRNA modifications during HIV-1 infection**. *A*, the host tRNAome (tRNA variants, modification levels (*red spheres*)) is altered upon HIV-1 infection. The expression and stability of certain tRNAs, their modifications, modification stoichiometries and/or subcellular localization may be impacted, altering the tRNA pool in the cell. tRNA^Phe^, whose wybutosine (yW) levels at position 37 are known to decrease during HIV-1 infection is boxed in *red*. *B*, SLFN-mediated tRNA cleavage. Codon specific tRNA cleavage by SLFN following HIV infection may be impacted by the modifications on tRNAs. *C*, impact of changes in the tRNAome on the protein landscape. With the host tRNA pool being reprogrammed (Fig. 4, step A), steps of protein synthesis at the ribosome (translation speed, fidelity, *etc*.) may be altered, leading to changes in the host proteome. The reprogrammed host proteome may have effects on viral replication. Viral/host protein synthesis may also be impacted by the changes in the tRNA pool, likely in a codon-usage dependent manner. *D*, programmed ribosomal frameshifting (PRF) events mediated by hypomodified tRNA. tRNA^Phe^ (*pink*) with reduced yW37 levels promotes frameshifting at the U_UUU_UUA slippery sequence. This helps maintain the optimal translation ratio of HIV-1 Gag protein with MA (matrix), CA (capsid), NC (nucleocapsid) and p6 domains and Gag-Pol (polymerase) polyprotein. *E*, tRNAs with specific modification patterns may be recruited to be packaged into virion particles. Since modifications on primer tRNA^Lys,3^ are important for reverse transcription, tRNA primers containing modifications may be selectively recruited to HIV-1 virions.

Despite four decades of research on HIV-1, relatively little is known about regulation of the tRNA/tDR pool and/or modifications in response to viral infection. While a full characterization of the tRNAome during HIV-1 infection is currently lacking, based on the substantial difference in codon usage between early and late HIV-1 proteins, changes in the tRNA pool are proposed to occur during viral infection (Fig. 4*A*) (46, 53). An anti-viral response that involves codon-usage-based tRNA cleavage by SLFN family of endonucleases has been reported in HIV-1 (Fig. 4*B*) (73). The impact of tRNA modifications on this cleavage process is unclear.

Since HIV-1 prefers A-ending codons and the tRNA pool in virions positively correlates with viral codon bias, modification levels at U34 are also likely regulated to maintain optimal viral translation (Fig. 4*C*) (53). With PRF being a crucial element in HIV-1 protein synthesis, yW37 downregulation in tRNA^Phe^(GAA) has functional implications for frameshifting (Fig. 4*D*) (109). However, the expression/activity changes in the corresponding tRNA-modifying enzymes (or erasers) and their mechanisms of regulation are unknown.

Specific modifications in the primer tRNA^Lys,3^ are proposed to facilitate reverse transcription; thus, mechanisms to ensure selection and packaging of properly modified tRNA primers likely exist (Fig. 4*E*). Interestingly, AC modifications that are involved in tRNA primer-template interactions outside of the PBS in reverse transcription initiation, mcm^5^s^2^U34 and ms^2^t^6^A37, are also recognized during LysRS binding to tRNA^Lys,3^ (129). This may ensure that tRNA^Lys,3^ containing AC modifications, is packaged into virions. Indeed, ms^2^t^6^A has been detected in tRNAs packaged into HIV-1 virus particles (126).

The m^1^A58 modification supports T-cell proliferation, as discussed above (89). Because HIV-1 preferentially infects activated T-cells, and m^1^A58 in tRNA^Lys,3^ is known to play a specific role in viral reverse transcription, it is possible that viral infection also upregulates this modification; this hypothesis remains to be tested. Moreover, m^1^A58 and other TΨC loop modifications participate in a broader modification circuit that may be collectively upregulated upon HIV-1 infection. Such coordinated regulation could help to ensure that the packaged tRNA^Lys,3^ carries the necessary TΨC-loop modifications to function as an effective stop during plus-strand synthesis in reverse transcription (Fig. 3*B*) (177, 180).

## tRNA-modifying enzymes (writers) and erasers in viral infections

A change in the tRNA modification landscape induced by viral infection suggests modulation of expression, stability, and/or activity of modifying enzymes (writers), erasers, and regulatory pathways. Upregulation of the KIAA1456 modifying enzyme, leading to tRNA modification changes upon CHIKV infection, discussed previously, is an example of such a scenario (55). Similarly, during SARS-CoV-2 infection, the expression levels of certain modifying enzymes were found to be modulated, correlating with changes in tRNA modification levels (60). Human tRNA methyltransferase 1 (TRMT1) that adds *N2*, *N2*-dimethylguanosine (m^2,2^G) at position 26 in tRNA is recognized and cleaved by the SARS-CoV-2 main protease/non-structural protein 5 (M^Pro^/Nsp5), leading to a reduction in m^2,2^G levels following infection (181, 182, 183, 184). However, viral replication is negatively impacted by TRMT1 knockout. Thus, TRMT1 levels are likely maintained at an optimal level that supports viral infection in host cells (182).

The *TRMT6* gene of the TRMT6-TRMT61A methyltransferase complex that incorporates m^1^A58 in tRNA was identified as one of the 311 host factors required for HIV-1 replication in a genome-scale RNAi screen (185). This agrees with the role of this modification in strand-transfer events during reverse transcription. An additional role of this writer complex on viral replication was described by Fukuda *et al*.; HIV-1 infection of CRISPR-generated *TRMT6* mutant cells showed reduced viral particle production and decreased accumulation of HIV-1 proteins such as integrase, capsid, Tat, Gag and Gag-Pol, while HIV-1 RNA levels and global protein levels remained unchanged (177). Whether this effect is directly correlated with tRNA hypomodification or occurs through a different mechanism is unknown. TRMT6 was also identified as an important host cell factor in hepatitis C virus (HCV) replication (186). Intriguingly, opposing effects upon *TRMT6* gene silencing were observed in two cell lines—HCV protein levels were increased in Huh-7 cells and decreased in U20S cells. Whether TRMT6 influences HCV replication through modification of tRNA(s) or a different pathway is unclear.

A tRNA modification eraser promotes RSV replication through specific tDR-biogenesis (187, 188, 189, 190). tDRs derived from full length tRNA, are known to control gene expression, translation, cell stress responses and development (191). Upon RSV infection, m^1^A57 in tRNA^Glu^(CUC) was demethylated by ALKBH1, which promoted tRNA^Glu^(CUC) 5′ fragment production through angiogenin (ANG) cleavage. The 5′ tDR repressed target mRNA, promoting viral replication. As m^1^A57 is known to promote structural stability, demethylation likely destabilizes the tRNA, allowing for cleavage by ANG. While ALKBH1 expression was not increased following infection, whether its activity is upregulated remains to be tested. These findings uncover a novel strategy by which viruses exploit tRNA modification reversibility to their advantage.

## Modifications in virally encoded tRNAs and tRNA-like structures (TLSs)

Large DNA viruses such as bacteriophages, phycodnaviruses and mimiviruses encode their own tRNAs (192). *In silico* studies predict tRNA gene clusters in numerous viral genomes, including single-stranded (ss) and dsDNA viruses and ssRNA viruses (193). The functions of these tRNAs include supporting viral protein synthesis by matching viral codon usage and compensating for infection-induced changes in the host tRNAome (192, 194, 195). Some viral tRNAs were found to be aminoacylated (196), and a full complement of translation components, including tRNA-modifying enzymes—pseudouridine synthase, lysidine synthase, and RNA methyltransferase – have been identified in certain giant viral genomes (197, 198). Modifications on viral tRNAs may be involved in maintaining stability and structure, while expanding/restricting codon recognition.

A study of 18 tRNAs in T4-like Vibriophage demonstrated that viral tRNAs sustain translation in a dynamically-adapted phage codon-usage manner, as host DNA and RNA is degraded upon viral infection (195). Of the two phage-encoded tRNA(CAU)s, one contained the modifications 4-thiouridine (s^4^U) at position 8, 3-(3-amino-3-carboxypropyl)uridine (acp^3^U) at position 47, and 2-lysidine (k^2^C) at position 34. The presence of k^2^C34 expands the function of tRNA(CAU) from decoding only the AUG(Met) codon to also decoding AUA(Ile), diversifying the available tRNA pool as the host resources are depleted. 5-carboxymethylaminomethyl-2-thiouridine (cmnm^5^s^2^U) at position 34 on tRNA^Glu^ and m^1^G37 on most tRNA^Leu^ species were observed in both phage tRNAs and their host analogs, suggesting that phage tRNAs may be modified by the host tRNA-modifying enzymes (195).

Several positive-strand RNA plant viruses and a few animal viruses harbor TLSs at the 3′ end of their genomes (199). These RNA structures form a pseudoknotted aminoacyl-acceptor stem mimic that can be specifically aminoacylated with either Val, His or Tyr (192). TLSs are involved in viral RNA replication, stability, and genome packaging, preventing recombination and translation enhancement functions (199). The potential for TLSs to be modified was shown in tobacco mosaic virus (TMV) where, upon incubation with *E*. *coli* cell extracts, m^5^U was detected at the site where the modification is canonically present in the TψC arm of tRNAs (200, 201). Similarly, when the 3′ end of the turnip yellow mosaic virus (TYMV) RNA, which resembles the tRNA TψC arm, was incubated with yeast extract, modifications ψ65 and m^5^U38 (numbered from the 3′ end of viral RNA) were found in the TYMV TLS RNA; these positions are equivalent to ψ27 and m^5^U54 in tRNA, respectively (202). However, the 3′ end of TYMV RNA extracted from infected plant cells has been reported to contain no detectable levels of modified nucleosides (203). Conversely, in a replication enhancer sequence that mimics the TψC arm of tRNA^Asp^ in an intergenic region of brome mosaic virus RNA, the uridines that correspond to tRNA positions 54 and 55 contained the canonical modifications m^5^U and ψ, respectively (204). While mutations that disrupt TψC loop mimicry inhibit replication enhancer function, whether the modifications are essential for activity is unknown. The conserved body modifications involved in tRNA structure and folding may be necessary for TLSs to mimic canonical tRNA structure. The modifying enzymes that add these modifications and their mechanism of TLS recognition remain to be elucidated.

## tRNA modifications in anticodon nuclease-mediated antiviral defense

Cells ranging from unicellular bacteria to multicellular organisms employ different defense mechanisms against phage or viral infections. Anticodon nucleases (ACNases) are toxin proteins primarily found in unicellular organisms that perform endonucleolytic cleavage of specific tRNAs at the AC loop as a form of immune response against viral infection, cellular stress, or to kill competing organisms (205, 206, 207). This activity causes depletion of specific tRNAs in the cellular pool, arresting both viral and global cellular protein synthesis. Sequence and modifications in the AC loop determine selection and cleavage of specific tRNAs, driving this anti-viral defense mechanism (208, 209, 210). Here, we discuss three examples of how tRNA AC loop modifications are important in this anti-viral cellular immunity response in bacterial, unicellular eukaryotic, and human systems.

**Figure 5:**
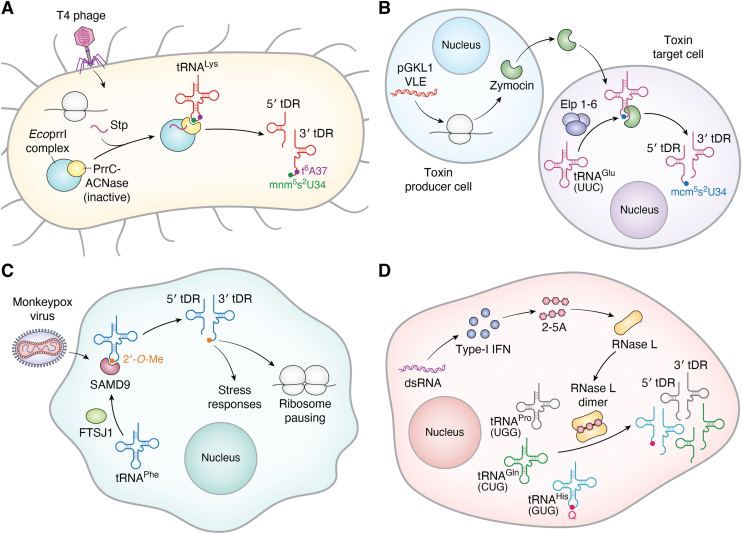
**tRNA modifications in anti-viral defense**. *A*, *E*. *coli* tRNA^Lys^ cleavage following T4 phage infection is mediated by AC loop modifications. T4 phage infection of *E*. *coli* causes PrrC-ACNase (*yellow*) to be activated through the binding of Stp peptide (*purple*). PrrC-ACNase activity is masked under normal conditions by EcoprrI complex (*blue*). Active PrrC-ACNase cleaves tRNA^Lys^ 5′ to U34 through recognition of mnm^5^s^2^U34 and t^6^A37 modifications, generating tRNA fragments. *B*, action of zymocin ACNase from pGKL1 virus-like element (VLE) in *Kluyveromyces lactis* requires wobble base modification. pGKL1 VLE in toxin producer cells produces zymocin (*green*), which is extracellularly secreted. When taken up by target cells, zymocin performs its killer ACNase function by cleaving tRNA^Glu^(UUC) between mcm^5^s^2^U34 and U35. Elp 1-6 protein complex (*purple*), which is involved in mcm^5^s^2^U34 synthesis, is required for zymocin function. *C*, monkeypox virus infection activates SAMD9 ACNase leading to specific cleavage of tRNA^Phe^ recognized by 2′-*O*-methylation at the wobble position. Upon monkeypox virus infection, SAMD9 protein (*maroon*) gets activated and cleaves tRNA^Phe^ at U34 through recognition of a unique 2′-*O*-ribose methylation in tRNA^Phe^, added by FTSJ1 (*green*). The resulting tRNA fragments cause downstream stress responses and ribosome pausing events. *D*, specific cleavage of tRNA^His^ by RNase L following immune activation is directed by Q34. Double-stranded RNA from viral infection triggers type-1 interferons (IFNs) (*blue*) to produce 2′-5′ oligoadenylate (2-5A). 2-5A binds to RNase L (*yellow*) causing its dimerization/activation. Activated RNase L cleaves tRNA^His^(GUG), tRNA^Pro^(UGG), and tRNA^Gln^(CUG), while Q34 in tRNA^His^(GUG) directs site-specific tRNA cleavage.

Upon T4 phage infection and expression of the phage peptide Stp in *E*. *coli*, PrrC-ACNase gets activated; this toxin’s cytotoxicity is otherwise masked by a type-I DNA restriction-modification (R-M) enzyme complex, EcoprrI (205, 211). Activated PrrC-ACNase recognizes and cleaves tRNA^Lys^ 5′ to the wobble base, generating 2′,3′-cyclic phosphate termini. This eventually causes death of infected host cells, preventing spread of phage (206). Unmodified *E*. *coli* tRNA^Lys^ is a less reactive substrate than the modified tRNA, and 5-methylaminomethyl-2-thiouridine (mnm^5^s^2^U34) and t^6^A37 in the AC loop significantly improve cleavage activity through direct contact of wobble base modification with PrrC residue Asp^287^ (Fig. 5*A*) (208, 209).

Certain yeast species harbor cytoplasmic linear dsDNA that encodes its own replication and transcription machineries called virus-like elements (VLE), which are phylogenetically related to infective viruses (207). VLEs such as *Kluyveromyces lactis* pGKL1 and *Pichia acacia* pPac1-2 produce toxins zymocin and PaT, respectively. These proteins are extracellularly secreted and encode subunits responsible for chitin binding and chitinase activity as well as hydrophobic domains, which allow transmembrane passage into target cells, where they perform killer ACNase function. Zymocin targets tRNA^Glu^(UUC) and cleaves between nt 34 and 35. Elp1-6 is involved in mcm^5^s^2^U synthesis at nt 34 and is indispensable for zymocin action, highlighting the importance of the wobble base modification for enzyme selectivity and efficiency (Fig. 5*B*). PaT performs its ACNase activity on tRNA^Gln^(UUG) through anticodon selectivity and requires the presence of a modification at either U32 or U34 (207).

Human sterile alpha motif domain-containing 9 (SAMD9), an immune sensor and potent restriction factor for poxviruses such as monkeypox virus and vaccinia virus, was recently reported to be an ACNase (210). Whereas ACNase activity of SAMD9 is latent under normal conditions, poxvirus infection activates an N-terminal effector domain in SAMD9 to specifically cleave tRNA^Phe^ at its wobble position to yield 3′-OH and 5′-phosphate termini. tRNA specificity is conferred by a unique 2′-*O*-ribose methylation at nt 34 in eukaryotic tRNA^Phe^, which is added by the RNA methyltransferase FTSJ1. This cleavage leads to Phe codon-specific ribosomal pausing and stress responses, resulting in inhibition of protein synthesis and viral replication (Fig. 5*C*) (210).

## tRNA modifications in RNase L-mediated anti-viral defense

Targeted tRNA cleavage also occurs in response to dsRNA production as a result of viral infection in mammalian cells (212, 213, 214). Viral dsRNA activates the innate immune system triggering type-I interferons (IFNs), which leads to synthesis of 2′-5′-linked oligoadenylates (2-5A). Binding of 2-5A activates RNase L through dimerization, resulting in cleavage of small noncoding RNAs such as tRNAs and Y-RNAs at UNˆN consensus sites (^ˆ^site of cleavage). tRNA^His^(GUG), tRNA^Pro^(UGG), and tRNA^Gln^(CUG) are specifically cleaved in the AC stemloop. While tRNA^His^ contains three putative sites of cleavage in the AC loop (3′ to nt 33, 34 and 36), only one site (nt 36) is specifically cleaved *in vivo*. However, all three products were found upon *in vitro* synthesized tRNA^His^ cleavage (Fig. 5*D*). The presence of a bulky Q modification at nt 34 was found to abrogate cleavage at non-specific positions and maintain RNase L specificity, indicating that modifications in the AC loop determine tRNA as well as nt specificity of RNase L (212). Although a global arrest in protein synthesis following RNase L activation could not be attributed to a decrease in tRNA levels, the possibility that tRNA cleavage products may engage in downstream translation inhibition pathways cannot be excluded. Indeed, a recent study reported that tDR generated from RNase L-mediated cleavage of tRNA, inhibited translation and generated an innate immune-like response to viral infection (215).

## Summary and outlook

Once considered passive chemical decorations, tRNA modifications are now widely recognized as key regulators of gene expression at the post-transcriptional level, particularly under changing cellular or environmental conditions. During viral infection, both tRNAs and their modification levels are actively reprogrammed—not only to modulate translation efficiency but also to fulfill specialized roles in facilitating or restricting viral replication. Until recently, our detailed knowledge of tRNA landscape changes and functions was limited, mainly due to limitations in tRNA sequencing and modification detection tools (see Kompatscher *et al*. for an excellent review) (216). The recent resurgence of interest in tRNA modification biology has growing implications for virology. The study of viral tRNA epitranscriptomics is still in its early stages but will benefit from insights gained in other fields such as cancer biology (217).

Changes in modifications can be either virally induced or be the result of the host immune response, which adds an additional layer of complexity. A comprehensive characterization of the tRNAome during viral infection, including tRNA abundance, modification levels and fragmentation with temporal dissection, is needed. Care should be taken in selecting the cell lines for such studies to ensure biological relevance, as tRNA profiles vary in a cell-type-dependent manner, as well as between tissues (218, 219). Numerous unresolved questions remain regarding the upstream triggers and downstream consequences of tRNAome changes during infection. For example, what causes the changes in specific tRNA modifications within select tRNA species, and are these changes stoichiometric? Do spatially distinct changes in tRNA modification profiles occur, including in tRNAs selectively packaged into viral particles? What mechanisms underlie the targeting of particular tRNA sequences, and are the expression levels of the corresponding modifying enzymes altered? Are these changes pro- or anti-viral and do tRNA modifications contribute functions apart from their canonical roles in translation? Continued advances in technology development are imperative to answer these questions. Direct RNA sequencing, such as Oxford nanopore technology (ONT), is a promising avenue to directly sequence tRNAs with information on their modification profile, either through base miscalling or direct analysis of the signal from the electric current (220, 221, 222). LC-MS/MS is a central tool for modification identification, which when combined with direct sequencing, provides robust information (223). Sequencing technologies that overcome challenges in tRNA sequencing (due to heavy modification levels, short sequence lengths, and stable tertiary structures) have significantly advanced the field (31, 224).

These advances will be critical in the development of antiviral therapeutic strategies targeting the tRNAome. Key tRNA molecules and/or modifications that play important roles in viral replication (*e*.*g*., m^1^A58 in human tRNA^Lys,3^ that serves as the +sss in HIV-1 reverse transcription) are potential targets with minimal impact on the overall host cell tRNA functional landscape. Targeting modifying enzymes that are temporally regulated during viral infection is another potential anti-viral therapeutic strategy. As the global tRNA pool is also modulated to promote viral codon-biased protein synthesis, delivering tRNA pools/tRNA expressing vectors to disrupt these overall changes could negatively impact viral protein production. This strategy might be particularly useful for disrupting PRF, as the AC modification profile of tRNAs at frameshifting sites is fine-tuned to ensure optimal viral protein expression. A comprehensive understanding of changes in the tRNA modification and cleavage landscape during viral infection, and the functional significance of these changes, will advance the development of antiviral therapeutics targeting the tRNAome.

## Data availability

All data generated or analyzed during this study are included in this published article and its supporting information files. Raw data is available upon request

## Conflict of interest

The authors declare that they do not have any conflicts of interest with the content of this article.

## References

[1] Doherty J., Guo M. (2016). Encylopedia of Cell Biology.

[2] Kirchner S., Ignatova Z. (2015). Emerging roles of tRNA in adaptive translation, signalling dynamics and disease. Nat. Rev. Genet..

[3] Huang H.Y., Hopper A.K. (2016). Multiple layers of stress-induced regulation in tRNA biology. Life (Basel).

[4] Zhang J. (2024). Recognition of the tRNA structure: everything everywhere but not all at once. Cell Chem. Biol..

[5] Phizicky E.M., Hopper A.K. (2023). The life and times of a tRNA. RNA.

[6] Agris P.F., Eruysal E.R., Narendran A., Vare V.Y.P., Vangaveti S., Ranganathan S.V. (2018). Celebrating wobble decoding: half a century and still much is new. RNA Biol..

[7] Crick F.H. (1966). Codon-anticodon pairing: the wobble hypothesis. J. Mol. Biol..

[8] Talló-Parra M., Muscolino E., Díez J. (2022). The host tRNA epitranscriptome: A new player in RNA virus infections. Front. Virol..

[9] Cui W., Zhao D., Jiang J., Tang F., Zhang C., Duan C. (2023). tRNA modifications and modifying enzymes in disease, the potential therapeutic targets. Int. J. Biol. Sci..

[10] Liang E., Wang W., Zhang L. (2025). Decoding tRNA dynamics in neuroimmune disorders: mechanistic insights, diagnostic innovations, and therapeutic opportunities. Front. Immunol..

[11] Gu C., Begley T.J., Dedon P.C. (2014). tRNA modifications regulate translation during cellular stress. FEBS Lett..

[12] Suzuki T. (2021). The expanding world of tRNA modifications and their disease relevance. Nat. Rev. Mol. Cell Biol..

[13] Wang L., Lin S. (2023). Emerging functions of tRNA modifications in mRNA translation and diseases. J. Genet. Genomics..

[14] Zhou Z., Sun B., Yu D., Bian M. (2021). Roles of tRNA metabolism in aging and lifespan. Cell Death Dis..

[15] Smith T.J., Giles R.N., Koutmou K.S. (2023). Anticodon stem-loop tRNA modifications influence codon decoding and frame maintenance during translation. Semin. Cell Dev. Biol..

[16] Yared M.J., Marcelot A., Barraud P. (2024). Beyond the anticodon: tRNA core modifications and their impact on structure, translation and stress adaptation. Genes (Basel).

[17] Zhang W., Foo M., Eren A.M., Pan T. (2022). tRNA modification dynamics from individual organisms to metaepitranscriptomics of microbiomes. Mol. Cell.

[18] Ohira T., Minowa K., Sugiyama K., Yamashita S., Sakaguchi Y., Miyauchi K. (2022). Reversible RNA phosphorylation stabilizes tRNA for cellular thermotolerance. Nature.

[19] Aharon-Hefetz N., Frumkin I., Mayshar Y., Dahan O., Pilpel Y., Rak R. (2020). Manipulation of the human tRNA pool reveals distinct tRNA sets that act in cellular proliferation or cell cycle arrest. Elife.

[20] Schultz S.K., Kothe U. (2024). RNA modifying enzymes shape tRNA biogenesis and function. J. Biol. Chem..

[21] Mitchener M.M., Begley T.J., Dedon P.C. (2023). Molecular coping mechanisms: reprogramming tRNAs to regulate codon-biased translation of stress response proteins. Acc. Chem. Res..

[22] Gunaratne L., Moore H., Albaum N., Casius A., Henderson J., Kessler A. (2025). Key RNA-binding domains in the La protein establish tRNA modification levels in Trypanosoma brucei. Nucleic Acids Res..

[23] Ando D., Rashad S., Begley T.J., Endo H., Aoki M., Dedon P.C. (2025). Decoding codon bias: the role of tRNA modifications in tissue-specific translation. Int. J. Mol. Sci..

[24] Pollo-Oliveira L., de Crecy-Lagard V. (2019). Can protein expression be regulated by modulation of tRNA modification profiles?. Biochemistry.

[25] Torrent M., Chalancon G., de Groot N.S., Wuster A., Babu M.M. (2018). Cells alter their tRNA abundance to selectively regulate protein synthesis during stress conditions. Sci. Signal..

[26] Chan C.T., Dyavaiah M., DeMott M.S., Taghizadeh K., Dedon P.C., Begley T.J. (2010). A quantitative systems approach reveals dynamic control of tRNA modifications during cellular stress. PLoS Genet..

[27] Helm M., Motorin Y. (2017). Detecting RNA modifications in the epitranscriptome: predict and validate. Nat. Rev. Genet..

[28] Limbach P.A., Paulines M.J. (2017). Going global: the new era of mapping modifications in RNA. Wiley Interdiscip. Rev. RNA.

[29] Thakur P., Estevez M., Lobue P.A., Limbach P.A., Addepalli B. (2020). Improved RNA modification mapping of cellular non-coding RNAs using C- and U-specific RNases. Analyst.

[30] Schwartz S., Motorin Y. (2017). Next-generation sequencing technologies for detection of modified nucleotides in RNAs. RNA Biol..

[31] Padhiar N.H., Katneni U., Komar A.A., Motorin Y., Kimchi-Sarfaty C. (2024). Advances in methods for tRNA sequencing and quantification. Trends Genet..

[32] Zhang J. (2021). Interplay between host tRNAs and HIV-1: A structural perspective. Viruses.

[33] Proud C.G. (2007). Signalling to translation: how signal transduction pathways control the protein synthetic machinery. Biochem. J..

[34] Jung H., Gkogkas C.G., Sonenberg N., Holt C.E. (2014). Remote control of gene function by local translation. Cell.

[35] Dufner A., Thomas G. (1999). Ribosome S6 kinase signaling and the control of translation. Exp. Cell Res..

[36] Hershey J.W. (1989). Protein phosphorylation controls translation rates. J. Biol. Chem..

[37] Morris D.R. (1995). Growth control of translation in mammalian cells. Prog. Nucleic Acid Res. Mol. Biol..

[38] Njenga R., Boele J., Ozturk Y., Koch H.G. (2023). Coping with stress: how bacteria fine-tune protein synthesis and protein transport. J. Biol. Chem..

[39] Williams T.D., Rousseau A. (2024). Translation regulation in response to stress. FEBS J..

[40] Wek R.C. (2018). Role of eIF2alpha kinases in translational control and adaptation to cellular stress. Cold Spring Harb. Perspect. Biol..

[41] Saikia M., Wang X., Mao Y., Wan J., Pan T., Qian S.B. (2016). Codon optimality controls differential mRNA translation during amino acid starvation. RNA.

[42] Jan E., Mohr I., Walsh D. (2016). A cap-to-tail guide to mRNA translation strategies in virus-infected cells. Annu. Rev. Virol..

[43] Walsh D., Mathews M.B., Mohr I. (2013). Tinkering with translation: protein synthesis in virus-infected cells. Cold Spring Harb. Perspect. Biol..

[44] Hoang H.D., Neault S., Pelin A., Alain T. (2021). Emerging translation strategies during virus-host interaction. Wiley Interdiscip. Rev. RNA.

[45] Stern-Ginossar N., Thompson S.R., Mathews M.B., Mohr I. (2019). Translational control in virus-infected cells. Cold Spring Harb. Perspect. Biol..

[46] Li M., Kao E., Gao X., Sandig H., Limmer K., Pavon-Eternod M. (2012). Codon-usage-based inhibition of HIV protein synthesis by human schlafen 11. Nature.

[47] Parvathy S.T., Udayasuriyan V., Bhadana V. (2022). Codon usage bias. Mol. Biol. Rep..

[48] Rocha E.P. (2004). Codon usage bias from tRNA's point of view: redundancy, specialization, and efficient decoding for translation optimization. Genome Res..

[49] Carbone A. (2008). Codon bias is a major factor explaining phage evolution in translationally biased hosts. J. Mol. Evol..

[50] Lucks J.B., Nelson D.R., Kudla G.R., Plotkin J.B. (2008). Genome landscapes and bacteriophage codon usage. PLoS Comput. Biol..

[51] Bahir I., Fromer M., Prat Y., Linial M. (2009). Viral adaptation to host: a proteome-based analysis of codon usage and amino acid preferences. Mol. Syst. Biol..

[52] Pavesi A., Romerio F. (2023). Different patterns of codon usage and amino acid composition across primate lentiviruses. Viruses.

[53] van Weringh A., Ragonnet-Cronin M., Pranckeviciene E., Pavon-Eternod M., Kleiman L., Xia X. (2011). HIV-1 modulates the tRNA pool to improve translation efficiency. Mol. Biol. Evol..

[54] Chan C., Pham P., Dedon P.C., Begley T.J. (2018). Lifestyle modifications: coordinating the tRNA epitranscriptome with codon bias to adapt translation during stress responses. Genome Biol..

[55] Jungfleisch J., Bottcher R., Tallo-Parra M., Perez-Vilaro G., Merits A., Novoa E.M. (2022). CHIKV infection reprograms codon optimality to favor viral RNA translation by altering the tRNA epitranscriptome. Nat. Commun..

[56] Jenkins G.M., Holmes E.C. (2003). The extent of codon usage bias in human RNA viruses and its evolutionary origin. Virus. Res..

[57] Eldin P., David A., Hirtz C., Battini J.L., Briant L. (2024). SARS-CoV-2 displays a suboptimal codon usage bias for efficient translation in human cells diverted by hijacking the tRNA epitranscriptome. Int. J. Mol. Sci..

[58] van der Kuyl A.C., Berkhout B. (2012). The biased nucleotide composition of the HIV genome: a constant factor in a highly variable virus. Retrovirology.

[59] Berkhout B., Grigoriev A., Bakker M., Lukashov V.V. (2002). Codon and amino acid usage in retroviral genomes is consistent with virus-specific nucleotide pressure. AIDS Res. Hum. Retroviruses.

[60] Muscolino E., Puig-Torrents M., Buigues Bisquert J., Correa Mendonca D., Tallo-Parra M., Perez-Vilaro G. (2026). Coronaviruses reprogram the tRNA epitranscriptome to favor viral protein expression. Nat. Commun..

[61] Castellano L.A., McNamara R.J., Pallarés H.M., Gamarnik A.V., Alvarez D.E., Bazzini A.A. (2024). Dengue virus preferentially uses human and mosquito non-optimal codons. Mol. Syst. Biol..

[62] Kerkhofs K., Guydosh N.R., Bayfield M.A. (2025). Respiratory syncytial virus (RSV) enhances translation of virus-resembling AU-rich host transcripts. Virol. J..

[63] Lampi M., Gregorova P., Qasim M.S., Ahlblad N.C.V., Sarin L.P. (2023). Bacteriophage infection of the marine bacterium Shewanella glacialimarina induces dynamic changes in tRNA modifications. Microorganisms.

[64] Dremel S.E., Jimenez A.R., Tucker J.M. (2023). "Transfer" of power: the intersection of DNA virus infection and tRNA biology. Semin Cell Dev. Biol..

[65] Wang Y., Wang X., Avan A., Li P., Ou X., Pan Q. (2025). Coronaviruses remodel the mature human tRNAome to modulate infection. Virulence.

[66] Dremel S.E., Sivrich F.L., Tucker J.M., Glaunsinger B.A., DeLuca N.A. (2022). Manipulation of RNA polymerase III by Herpes Simplex Virus-1. Nat. Commun..

[67] Tucker J.M., Schaller A.M., Willis I., Glaunsinger B.A. (2020). Alteration of the premature tRNA landscape by gammaherpesvirus infection. mBio.

[68] Pavon-Eternod M., David A., Dittmar K., Berglund P., Pan T., Bennink J.R. (2013). Vaccinia and influenza A viruses select rather than adjust tRNAs to optimize translation. Nucleic Acids Res..

[69] Hernandez-Alias X., Benisty H., Schaefer M.H., Serrano L. (2021). Translational adaptation of human viruses to the tissues they infect. Cell Rep..

[70] Santos M., Fidalgo A., Varanda A.S., Oliveira C., Santos M.A.S. (2019). tRNA deregulation and its consequences in cancer. Trends Mol. Med..

[71] Li Y., Zhang R., Wang Y., Li P., Li Y., Janssen H.L.A. (2023). Hepatitis E virus infection remodels the mature tRNAome in macrophages to orchestrate NLRP3 inflammasome response. Proc. Natl. Acad. Sci. U. S. A..

[72] Valenzuela C., Saucedo S., Llano M. (2024). Schlafen14 impairs HIV-1 expression in a codon usage-dependent manner. Viruses.

[73] Kobayashi-Ishihara M., Frazao Smutna K., Alonso F.E., Argilaguet J., Esteve-Codina A., Geiger K. (2023). Schlafen 12 restricts HIV-1 latency reversal by a codon-usage dependent post-transcriptional block in CD4+ T cells. Commun. Biol..

[74] Li M., Kao E., Malone D., Gao X., Wang J.Y.J., David M. (2018). DNA damage-induced cell death relies on SLFN11-dependent cleavage of distinct type II tRNAs. Nat. Struct. Mol. Biol..

[75] Lee S., Hoyt S., Wu X., Garvie C., McGaunn J., Shekhar M. (2023). Velcrin-induced selective cleavage of tRNA(Leu)(TAA) by SLFN12 causes cancer cell death. Nat. Chem. Biol..

[76] Metzner F.J., Wenzl S.J., Kugler M., Krebs S., Hopfner K.P., Lammens K. (2022). Mechanistic understanding of human SLFN11. Nat. Commun..

[77] Yang J.Y., Deng X.Y., Li Y.S., Ma X.C., Feng J.X., Yu B. (2018). Structure of Schlafen13 reveals a new class of tRNA/rRNA- targeting RNase engaged in translational control. Nat. Commun..

[78] McIntyre W., Netzband R., Bonenfant G., Biegel J.M., Miller C., Fuchs G. (2018). Positive-sense RNA viruses reveal the complexity and dynamics of the cellular and viral epitranscriptomes during infection. Nucleic Acids Res..

[79] Su D., Chan C.T., Gu C., Lim K.S., Chionh Y.H., McBee M.E. (2014). Quantitative analysis of ribonucleoside modifications in tRNA by HPLC-coupled mass spectrometry. Nat. Protoc..

[80] Kitamura A., Nishimoto M., Sengoku T., Shibata R., Jager G., Bjork G.R. (2012). Characterization and structure of the Aquifex aeolicus protein DUF752: a bacterial tRNA-methyltransferase (MnmC2) functioning without the usually fused oxidase domain (MnmC1). J. Biol. Chem..

[81] Chan C., Kwan Sze N.S., Suzuki Y., Ohira T., Suzuki T., Begley T.J. (2023). Dengue Virus exploits the host tRNA epitranscriptome to promote viral replication. bioRxiv.

[82] Aharon-Hefetz N., Schwartz M., Aharon E., Stern-Ginossar N., Dahan O., Pilpel Y. (2026). Essentiality and dynamic expression of the human tRNA pool during viral infection. Mol. Syst. Biol..

[83] Nagayoshi Y., Nishiguchi K., Yamamura R., Chujo T., Oshiumi H., Nagata H. (2022). t(6)A and ms(2)t(6)A modified nucleosides in serum and urine as strong candidate biomarkers of COVID-19 infection and severity. Biomolecules.

[84] Zhang W., Westhof E. (2025). The biology of tRNA t(6)A modification and hypermodifications-biogenesis and disease relevance. J. Mol. Biol..

[85] Katanski C.D., Alshammary H., Watkins C.P., Huang S., Gonzales-Reiche A., Sordillo E.M. (2022). tRNA abundance, modification and fragmentation in nasopharyngeal swabs as biomarkers for COVID-19 severity. Front. Cell Dev. Biol..

[86] Rak R., Polonsky M., Eizenberg-Magar I., Mo Y., Sakaguchi Y., Mizrahi O. (2021). Dynamic changes in tRNA modifications and abundance during T cell activation. Proc. Natl. Acad. Sci. U. S. A..

[87] Schaffrath R., Leidel S.A. (2017). Wobble uridine modifications-a reason to live, a reason to die?. RNA Biol..

[88] Jitobaom K., Phakaratsakul S., Sirihongthong T., Chotewutmontri S., Suriyaphol P., Suptawiwat O. (2020). Codon usage similarity between viral and some host genes suggests a codon-specific translational regulation. Heliyon.

[89] Liu Y., Zhou J., Li X., Zhang X., Shi J., Wang X. (2022). tRNA-m(1)A modification promotes T cell expansion via efficient MYC protein synthesis. Nat. Immunol..

[90] Nedialkova D.D., Leidel S.A. (2015). Optimization of codon translation rates via tRNA modifications maintains proteome integrity. Cell.

[91] Rezgui V.A., Tyagi K., Ranjan N., Konevega A.L., Mittelstaet J., Rodnina M.V. (2013). tRNA tKUUU, tQUUG, and tEUUC wobble position modifications fine-tune protein translation by promoting ribosome A-site binding. Proc. Natl. Acad. Sci. U. S. A..

[92] Yarian C., Marszalek M., Sochacka E., Malkiewicz A., Guenther R., Miskiewicz A. (2000). Modified nucleoside dependent Watson-Crick and wobble codon binding by tRNALys UUU species. Biochemistry.

[93] Hogan C.A., Gratz S.J., Dumouchel J.L., Thakur R.S., Delgado A., Lentini J.M. (2023). Expanded tRNA methyltransferase family member TRMT9B regulates synaptic growth and function. EMBO Rep..

[94] Gu C., Ramos J., Begley U., Dedon P., Fu D., Begley T. (2018). Phosphorylation of human TRM9L integrates multiple stress-signaling pathways for tumor growth suppression. Sci. Adv..

[95] Begley U., Sosa M.S., Avivar-Valderas A., Patil A., Endres L., Estrada Y. (2013). A human tRNA methyltransferase 9-like protein prevents tumour growth by regulating LIN9 and HIF1-alpha *EMBO*. Mol. Med..

[96] Begley U., Dyavaiah M., Patil A., Rooney J.P., DiRenzo D., Young C.M. (2007). Trm9-catalyzed tRNA modifications link translation to the DNA damage response. Mol. Cell.

[97] Eldin P., Bernard E., Vågbø C.B., George L., Ajiro M., Hagiwara M. (2025). Zika Virus Reprograms the Host tRNA Epitranscriptome to Adapt Translation to A-ending Codon Bias. bioRxiv.

[98] Eldin P., Briant L. (2025). tRNA modifications: a tale of two viruses-SARS-CoV-2 and ZIKV. Int. J. Mol. Sci..

[99] Hill C.H., Brierley I. (2023). Structural and functional insights into viral programmed ribosomal frameshifting. Annu. Rev. Virol..

[100] Champagne J., Mordente K., Nagel R., Agami R. (2022). Slippy-sloppy translation: a tale of programmed and induced-ribosomal frameshifting. Trends Genet..

[101] Brierley I., Meredith M.R., Bloys A.J., Hagervall T.G. (1997). Expression of a coronavirus ribosomal frameshift signal in Escherichia coli: influence of tRNA anticodon modification on frameshifting. J. Mol. Biol..

[102] Brierley I., Dos Ramos F.J. (2006). Programmed ribosomal frameshifting in HIV-1 and the SARS-CoV. Virus. Res..

[103] Riegger R.J., Caliskan N. (2022). Thinking outside the frame: impacting genomes capacity by programmed ribosomal frameshifting. Front. Mol. Biosci..

[104] Marquet R., Henri Grosjean RB (1998). Modification and Editing of RNA.

[105] Shehu-Xhilaga M., Crowe S.M., Mak J. (2001). Maintenance of the Gag/gag-Pol ratio is important for human immunodeficiency virus type 1 RNA dimerization and viral infectivity. J. Virol..

[106] Dulude D., Berchiche Y.A., Gendron K., Brakier-Gingras L., Heveker N. (2006). Decreasing the frameshift efficiency translates into an equivalent reduction of the replication of the human immunodeficiency virus type 1. Virology.

[107] Caliskan N., Katunin V.I., Belardinelli R., Peske F., Rodnina M.V. (2014). Programmed -1 frameshifting by kinetic partitioning during impeded translocation. Cell.

[108] Chen J., Petrov A., Johansson M., Tsai A., O'Leary S.E., Puglisi J.D. (2014). Dynamic pathways of -1 translational frameshifting. Nature.

[109] Hatfield D., Feng Y.-X., Lee B.L., Rein A., Levin J.G., Oroszlan S. (1989). Chromatographic analysis of the aminoacyl-tRNAs which are required for translation of codons at and around the ribosomal frameshift sites of HIV, HTLV-1, and BLV. Virology.

[110] Urbonavicius J., Qian Q., Durand J.M., Hagervall T.G., Bjork G.R. (2001). Improvement of reading frame maintenance is a common function for several tRNA modifications. EMBO J..

[111] Carlson B.A., Kwon S.Y., Chamorro M., Oroszlan S., Hatfield D.L., Lee B.J. (1999). Transfer RNA modification status influences retroviral ribosomal frameshifting. Virology.

[112] Carlson B.A., Kwon S.Y., Lee B.J., Hatfield D. (2000). Yeast asparagine (Asn) tRNA without Q base promotes eukaryotic frameshifting more efficiently than mammalian Asn tRNAs with or without Q base. Mol. Cells.

[113] Carlson B.A., Lee B.J., Hatfield D.L. (2008). Ribosomal frameshifting in response to hypomodified tRNAs in Xenopus oocytes. Biochem. Biophys. Res. Commun..

[114] Marczinke B., Hagervall T.G., Brierley I. (2000). The Q-base of asparaginyl-tRNA is dispensable for efficient -1 ribosomal frameshifting in eukaryotes. J. Mol. Biol..

[115] Waas W.F., Druzina Z., Hanan M., Schimmel P. (2007). Role of a tRNA base modification and its precursors in frameshifting in eukaryotes. J. Biol. Chem..

[116] Rossello-Tortella M., Llinas-Arias P., Sakaguchi Y., Miyauchi K., Davalos V., Setien F. (2020). Epigenetic loss of the transfer RNA-modifying enzyme TYW2 induces ribosome frameshifts in colon cancer. Proc. Natl. Acad. Sci. U. S. A..

[117] Korniy N., Goyal A., Hoffmann M., Samatova E., Peske F., Pohlmann S. (2019). Modulation of HIV-1 Gag/gag-pol frameshifting by tRNA abundance. Nucleic Acids Res..

[118] Bjork G.R., Wikstrom P.M., Bystrom A.S. (1989). Prevention of translational frameshifting by the modified nucleoside 1-methylguanosine. Science.

[119] Masuda I., McGuigan H., Maharjan S., Yamaki Y., Hou Y.M. (2025). Connecting tRNA charging and decoding through the axis of nucleotide modifications at position 37. J. Mol. Biol..

[120] Kimbrough E.M., Nguyen H.A., Li H., Mattingly J.M., Bailey N.A., Ning W. (2025). An RNA modification prevents extended codon-anticodon interactions from facilitating +1 frameshifting. Nat. Commun..

[121] Carlson B.A., Mushinski J.F., Henderson D.W., Kwon S.Y., Crain P.F., Lee B.J. (2001). 1-Methylguanosine in place of Y base at position 37 in phenylalanine tRNA is responsible for its shiftiness in retroviral ribosomal frameshifting. Virology.

[122] Maynard N.D., Macklin D.N., Kirkegaard K., Covert M.W. (2012). Competing pathways control host resistance to virus via tRNA modification and programmed ribosomal frameshifting. Mol. Syst. Biol..

[123] Levin M.E., Hendrix R.W., Sherwood R.C. (1993). A programmed translational frameshift is required for the synthesis of a bacteriophage λ tail assembly protein. J. Mol. Biol..

[124] Eckwahl M.J., Telesnitsky A., Wolin S.L. (2016). Host RNA packaging by retroviruses: a newly synthesized story. mBio.

[125] Pena N., Zhang W., Watkins C., Halucha M., Alshammary H., Hernandez M.M. (2022). Profiling selective packaging of host RNA and viral RNA modification in SARS-CoV-2 viral preparations. Front. Cell Dev. Biol..

[126] Simonova A., Svojanovska B., Trylcova J., Hubalek M., Moravcik O., Zavrel M. (2019). LC/MS analysis and deep sequencing reveal the accurate RNA composition in the HIV-1 virion. Sci. Rep..

[127] Pavon-Eternod M., Wei M., Pan T., Kleiman L. (2010). Profiling non-lysyl tRNAs in HIV-1. RNA.

[128] Jin D., Musier-Forsyth K. (2019). Role of host tRNAs and aminoacyl-tRNA synthetases in retroviral replication. J. Biol. Chem..

[129] Devarkar S.C., Budding C.R., Pathirage C., Kavoor A., Herbert C., Limbach P.A. (2025). Structural basis for aminoacylation of cellular modified tRNALys3 by human lysyl-tRNA synthetase. Nucleic Acids Res..

[130] Keith G., Heyman T. (1990). Heterogeneities in vertebrate tRNAsTrp avian retroviruses package only as a primer the tRNATrp lacking modified m2G in positon 7. Nucleic Acids Res..

[131] Freund I., Eigenbrod T., Helm M., Dalpke A.H. (2019). RNA modifications modulate activation of innate toll-like receptors. Genes (Basel).

[132] Burdick R.C., Hu W.S., Pathak V.K. (2013). Nuclear import of APOBEC3F-labeled HIV-1 preintegration complexes. Proc. Natl. Acad. Sci. U. S. A..

[133] Burdick R.C., Li C., Munshi M., Rawson J.M.O., Nagashima K., Hu W.S. (2020). HIV-1 uncoats in the nucleus near sites of integration. Proc. Natl. Acad. Sci. U. S. A..

[134] Muller T.G., Zila V., Peters K., Schifferdecker S., Stanic M., Lucic B. (2021). HIV-1 Uncoating by release of viral cDNA from capsid-like structures in the nucleus of infected cells. Elife.

[135] Gifford L.B., Melikyan G.B. (2024). HIV-1 capsid uncoating is a multistep process that proceeds through defect formation followed by disassembly of the capsid lattice. ACS Nano.

[136] Kariko K., Buckstein M., Ni H., Weissman D. (2005). Suppression of RNA recognition by Toll-like receptors: the impact of nucleoside modification and the evolutionary origin of RNA. Immunity.

[137] Gehrig S., Eberle M.E., Botschen F., Rimbach K., Eberle F., Eigenbrod T. (2012). Identification of modifications in microbial, native tRNA that suppress immunostimulatory activity. J. Exp. Med..

[138] Jockel S., Nees G., Sommer R., Zhao Y., Cherkasov D., Hori H. (2012). The 2'-O-methylation status of a single guanosine controls transfer RNA-mediated toll-like receptor 7 activation or inhibition. J. Exp. Med..

[139] Keller P., Freund I., Marchand V., Bec G., Huang R., Motorin Y. (2018). Double methylation of tRNA-U54 to 2'-O-methylthymidine (Tm) synergistically decreases immune response by toll-like receptor 7. Nucleic Acids Res..

[140] Simonova A., Romanska V., Benoni B., Skubnik K., Smerdova L., Prochazkova M. (2022). Honeybee Iflaviruses pack specific tRNA fragments from host cells in their virions. Chembiochem.

[141] Bartuli J., Jungwirth S., Dixit M., Okuda T., Zimmermann J.P., Erlacher M. (2025). tRNA as an assembly chaperone for a macromolecular transcription-processing complex. Nat. Struct. Mol. Biol..

[142] Grimm C., Hillen H.S., Bedenk K., Bartuli J., Neyer S., Zhang Q. (2019). Structural basis of poxvirus transcription: vaccinia RNA polymerase complexes. Cell.

[143] Mak J., Kleiman L. (1997). Primer tRNAs for reverse transcription. J. Virol..

[144] Isel C., Ehresmann C., Marquet R. (2010). Initiation of HIV reverse transcription. Viruses.

[145] Feng Y.-X., Campbell S., Harvin D., Ehresmann B., Ehresmann C., Rein A. (1999). The human immunodeficiency virus type 1 gag polyprotein has nucleic acid chaperone activity: possible role in dimerization of genomic RNA and placement of tRNA on the primer binding site. J. Virol..

[146] Roldan A., Warren O.U., Russell R.S., Liang C., Wainberg M.A. (2005). A HIV-1 minimal gag protein is superior to nucleocapsid at in vitro annealing and exhibits multimerization-induced inhibition of reverse transcription. J. Biol. Chem..

[147] Jones C.P., Datta S.A., Rein A., Rouzina I., Musier-Forsyth K. (2011). Matrix domain modulates HIV-1 Gag's nucleic acid chaperone activity via inositol phosphate binding. J. Virol..

[148] Sleiman D., Goldschmidt V., Barraud P., Marquet R., Paillart J.C., Tisne C. (2012). Initiation of HIV-1 reverse transcription and functional role of nucleocapsid-mediated tRNA/viral genome interactions. Virus. Res..

[149] Marquet R., Dardel F. (2005). Fine-Tuning of RNA Functions by Modification and Editing.

[150] Aiyar A., Cobrinik D., Ge Z., Kung H.-J. (1992). Interaction between retroviral U5 RNA and the T4C loop of the tRNATrP primer is required for efficient initiation of reverse transcription. J. Virol..

[151] Larsen K.P., Mathiharan Y.K., Kappel K., Coey A.T., Chen D.H., Barrero D. (2018). Architecture of an HIV-1 reverse transcriptase initiation complex. Nature.

[152] Kang S.M., Morrow C.D. (1999). Genetic analysis of a unique human immunodeficiency virus type 1 (HIV-1) with a primer binding site complementary to tRNAMet supports a role for U5-PBS stem-loop RNA structures in initiation of HIV-1 reverse transcription. J. Virol..

[153] Isel C., Lanchy J.M., Le Grice S.F., Ehresmann C., Ehresmann B., Marquet R. (1996). Specific initiation and switch to elongation of human immunodeficiency virus type 1 reverse transcription require the post-transcriptional modifications of primer tRNA3Lys. EMBO J..

[154] Isel C., Marquet R., Keith G., Ehresmann C., Ehresmann B. (1993). Modified nucleotides of tRNALys3 modulate primer/template loop-loop interaction in the initiation complex of HIV-1 reverse transcription. J. Biol. Chem..

[155] Huang Y., Shalom A., Li Z., Wang J., Mak J., Wainberg M.A. (1996). Effects of modifying the tRNA3Lys anticodon on the initiation of human immunodeficiency virus type 1 reverse transcription. J. Virol..

[156] Liang C., Rong L., Gotte M., Li X., Quan Y., Kleiman L. (1998). Mechanistic studies of early pausing events during initiation of HIV-1 reverse transcription. J. Biol. Chem..

[157] Wakefield J.K., Kang S., Morrow C.D. (1996). Construction of a type 1 human immunodeficiency virus that maintains a primer binding site complementary to tRNAHis. J. Virol..

[158] Bajji A.C., Sundaram M., Myszka D.G., Davis D.R. (2002). An RNA complex of the HIV-1 A-loop and tRNA(Lys,3) is stabilized by nucleoside modifications. J. Am. Chem. Soc..

[159] Tisne C., Rigourd M., Marquet R., Ehresmann C., Dardel F. (2000). NMR and biochemical characterization of recombinant human tRNA3Lys expressed in Escherichia coli: identification of posttranscriptional nucleotide modifications required for efficient initiation of HIV-1 reverse transcription. RNA.

[160] Graham W.D., Barley-Maloney L., Stark C.J., Kaur A., Stolarchuk C., Sproat B. (2011). Functional recognition of the modified human tRNALys3(UUU) anticodon domain by HIV's nucleocapsid protein and a peptide mimic. J. Mol. Biol..

[161] Spears J.L., Xiao X., Hall C.K., Agris P.F. (2014). Amino acid signature enables proteins to recognize modified tRNA. Biochemistry.

[162] Beerens N., Groot F., Berkhout B. (2001). Initiation of HIV-1 reverse transcription is regulated by a primer activation signal. J. Biol. Chem..

[163] Beerens N., Klaver B., Berkhout B. (2000). A structured RNA motif is involved in correct placement of the tRNA3Lys primer onto the human immunodeficiency virus genome. J. Virol..

[164] Sleiman D., Barraud P., Brachet F., Tisne C. (2013). The interaction between tRNA(Lys) 3 and the primer activation signal deciphered by NMR spectroscopy. PLoS One.

[165] Zhou H., Kimsey I.J., Nikolova E.N., Sathyamoorthy B., Grazioli G., McSally J. (2016). m(1)A and m(1)G disrupt A-RNA structure through the intrinsic instability of hoogsteen base pairs. Nat. Struct. Mol. Biol..

[166] Fosse P., Mougel M., Keith G., Westhof E., Ehresmann B., Ehresmann C. (1998). Modified nucleotides of tRNAPro restrict interactions in the binary primer/template complex of M-MuLV. J. Mol. Biol..

[167] Hu W.S., Hughes S.H. (2012). HIV-1 reverse transcription. Cold Spring Harb. Perspect. Med..

[168] Basu V.P., Song M., Gao L., Rigby S.T., Hanson M.N., Bambara R.A. (2008). Strand transfer events during HIV-1 reverse transcription. Virus. Res..

[169] Gilboa E., Mitra S.W., Goff S., Baltimore D. (1979). A detailed model of reverse transcription and tests of crucial aspects. Cell.

[170] Auxilien S., Keith G., Le Grice S.F.J., Darlix J. (1999). Role of post-transcriptional modifications of primer tRNALys,3 in the fidelity and efficacy of plus strand DNA transfer during HIV-1 reverse transcription. J. Biol. Chem..

[171] Zagryadskaya E.I., Doyon F.R., Steinberg S.V. (2003). Importance of the reverse hoogsteen base pair 54-58 for tRNA function. Nucleic Acids Res..

[172] Anderson J., Phan L., Cuesta R., Carlson B.A., Pak M., Asano K. (1998). The essential Gcd10p–Gcd14p nuclear complex is required for 1-methyladenosine modification and maturation of initiator methionyl-tRNA. Genes Dev..

[173] Ozanick S., Krecic A., Andersland J., Anderson J.T. (2005). The bipartite structure of the tRNA m1A58 methyltransferase from S. cerevisiae is conserved in humans. RNA.

[174] Wu T., Guo J., Bess J., Henderson L.E., Levin J.G. (1999). Molecular requirements for human immunodeficiency virus type 1 plus-strand transfer: analysis in reconstituted and endogenous reverse transcription systems. J. Virol..

[175] Burnett B.P., McHenry C.S. (1997). Posttranscriptional modification of retroviral primers is required for late stages ofDNA replication. Proc. Natl. Acad. Sci. U. S. A..

[176] Muthuswami R., Chen J., Burnett B.P., Thimmig R.L., Janjic N., McHenry C.S. (2002). The HIV plus-strand transfer reaction: determination of replication-competent intermediates and identification of a novel lentiviral element, the primer over-extension sequence. J. Mol. Biol..

[177] Fukuda H., Chujo T., Wei F.Y., Shi S.L., Hirayama M., Kaitsuka T. (2021). Cooperative methylation of human tRNA3Lys at positions A58 and U54 drives the early and late steps of HIV-1 replication. Nucleic Acids Res..

[178] Renda M.J., Rosenblatt J.D., Klimatcheva E., Demeter L.M., Bambara R.A., Planelles V. (2001). Mutation of the methylated tRNA(Lys)(3) residue A58 disrupts reverse transcription and inhibits replication of human immunodeficiency virus type 1. J. Virol..

[179] Smoczynski J., Yared M.J., Meynier V., Barraud P., Tisne C. (2024). Advances in the structural and functional understanding of m(1)A RNA modification. Acc. Chem. Res..

[180] Jones J.D., Franco M.K., Giles R.N., Eyler D.E., Tardu M., Smith T.J. (2024). Conserved 5-methyluridine tRNA modification modulates ribosome translocation. Proc. Natl. Acad. Sci. U. S. A..

[181] D'Oliviera A., Dai X., Mottaghinia S., Geissler E.P., Etienne L., Zhang Y. (2025). Recognition and cleavage of human tRNA methyltransferase TRMT1 by the SARS-CoV-2 main protease. eLife.

[182] Zhang K., Eldin P., Ciesla J.H., Briant L., Lentini J.M., Ramos J. (2024). Proteolytic cleavage and inactivation of the TRMT1 tRNA modification enzyme by SARS-CoV-2 main protease. elife.

[183] Lu J.L., Zhou X.L. (2023). SARS-CoV-2 main protease Nsp5 cleaves and inactivates human tRNA methyltransferase TRMT1. J. Mol. Cell Biol..

[184] Miczi M., Golda M., Kunkli B., Nagy T., Tozser J., Motyan J.A. (2020). Identification of host cellular protein substrates of SARS-COV-2 Main Protease. Int. J. Mol. Sci..

[185] Zhou H., Xu M., Huang Q., Gates A.T., Zhang X.D., Castle J.C. (2008). Genome-scale RNAi screen for host factors required for HIV replication. Cell Host Microbe.

[186] Jones D.M., Domingues P., Targett-Adams P., McLauchlan J. (2010). Comparison of U2OS and Huh-7 cells for identifying host factors that affect hepatitis C virus RNA replication. J. Gen. Virol..

[187] Choi E.J., Wu W., Zhang K., Yuan X., Deng J., Ismail D. (2022). Parent tRNA modification status determines the induction of functional tRNA-Derived RNA by respiratory syncytial virus infection. Viruses.

[188] Deng J., Ptashkin R.N., Chen Y., Cheng Z., Liu G., Phan T. (2015). Respiratory syncytial virus utilizes a tRNA fragment to suppress antiviral responses through a novel targeting mechanism. Mol. Ther..

[189] Wang Q., Lee I., Ren J., Ajay S.S., Lee Y.S., Bao X. (2013). Identification and functional characterization of tRNA-derived RNA fragments (tRFs) in respiratory syncytial virus infection. Mol. Ther..

[190] Zhou J., Liu S., Chen Y., Fu Y., Silver A.J., Hill M.S. (2017). Identification of two novel functional tRNA-derived fragments induced in response to respiratory syncytial virus infection. J. Gen. Virol..

[191] Muthukumar S., Li C.T., Liu R.J., Bellodi C. (2024). Roles and regulation of tRNA-derived small RNAs in animals. Nat. Rev. Mol. Cell Biol..

[192] Dreher T.W. (2010). Viral tRNAs and tRNA-like structures. Wiley Interdiscip. Rev. RNA.

[193] Morgado S., Vicente A.C. (2019). Global in-silico scenario of tRNA genes and their organization in virus genomes. Viruses.

[194] Bailly-Bechet M., Vergassola M., Rocha E. (2007). Causes for the intriguing presence of tRNAs in phages. Genome Res..

[195] Yang J.Y., Fang W., Miranda-Sanchez F., Brown J.M., Kauffman K.M., Acevero C.M. (2021). Degradation of host translational machinery drives tRNA acquisition in viruses. Cell Syst..

[196] Nishida K., Kawasaki T., Fujie M., Usami S., Yamada T. (1999). Aminoacylation of tRNAs encoded by chlorella virus CVK2. Virology.

[197] Schulz F., Yutin N., Ivanova N.N., Ortega D.R., Lee T.K., Vierheilig J. (2017). Giant viruses with an expanded complement of translation system components. Science.

[198] Abrahao J., Silva L., Silva L.S., Khalil J.Y.B., Rodrigues R., Arantes T. (2018). Tailed giant Tupanvirus possesses the most complete translational apparatus of the known virosphere. Nat. Commun..

[199] Wu S., Li X., Wang G. (2022). tRNA-like structures and their functions. FEBS J..

[200] Lesiewicz J., Dudock B. (1978). In vitro methylation of tobacco mosaic virus RNA with ribothymidine-forming tRNA methyltransferase of Escherichia coli. Biochim. Biophys. Acta.

[201] Marcu K., Dudock B. (1975). Methylation of TMV RNA. Biochem. Biophys. Res. Commun..

[202] Becker H.F., Motorin Y., Florentz C., Giege R., Grosjean H. (1998). Pseudouridine and ribothymidine formation in the tRNA-like domain of turnip yellow mosaic virus RNA. Nucleic Acids Res..

[203] Silberklang M., Prochiantz A., Haenni A.L., Rajbhandary U.L. (1977). Studies on the sequence of the 3'-terminal region of turnip-yellow-mosaic-virus RNA. Eur. J. Biochem..

[204] Baumstark T., Ahlquist P. (2001). The brome mosaic virus RNA3 intergenic replication enhancer folds to mimic a tRNA TCC-stem loop and is modified in vivo. RNA.

[205] Kaufmann G. (2000). Anticodon nucleases. Trends Biochem. Sci..

[206] Zhabokritsky A., Kutky M., Burns L.A., Karran R.A., Hudak K.A. (2011). RNA toxins: mediators of stress adaptation and pathogen defense. Wiley Interdiscip. Rev. RNA.

[207] Satwika D., Klassen R., Meinhardt F. (2012). Anticodon nuclease encoding virus-like elements in yeast. Appl. Microbiol. Biotechnol..

[208] Jiang Y., Meidler R., Amitsur M., Kaufmann G. (2001). Specific interaction between anticodon nuclease and the tRNA(Lys) wobble base. J. Mol. Biol..

[209] Jiang Y., Blanga S., Amitsur M., Meidler R., Krivosheyev E., Sundaram M. (2002). Structural features of tRNALys favored by anticodon nuclease as inferred from reactivities of anticodon stem and loop substrate analogs. J. Biol. Chem..

[210] Zhang F., Ji Q., Chaturvedi J., Morales M., Mao Y., Meng X. (2023). Human SAMD9 is a poxvirus-activatable anticodon nuclease inhibiting codon-specific protein synthesis. Sci. Adv..

[211] Levitz R., Chapman C., Amitsur M., Green R., Snyder L., Kaufmann G. (1990). The optional E.coli prr locus encodes a latent form of phage T4-induced anticodon nuclease. EMBO J..

[212] Donovan J., Rath S., Kolet-Mandrikov D., Korennykh A. (2017). Rapid RNase L-driven arrest of protein synthesis in the dsRNA response without degradation of translation machinery. RNA.

[213] Goldstein S.A., Thornbrough J.M., Zhang R., Jha B.K., Li Y., Elliott R. (2017). Lineage A Betacoronavirus NS2 proteins and the homologous Torovirus Berne pp1a carboxy-terminal domain are phosphodiesterases that antagonize activation of RNase L. J. Virol..

[214] Ireland D.D., Stohlman S.A., Hinton D.R., Kapil P., Silverman R.H., Atkinson R.A. (2009). RNase L mediated protection from virus induced demyelination. PLoS Pathog..

[215] Takenaka Y., Yamada A., Tomioka Y., Akiyama Y., Ivanov P. (2025). RNase L produces tRNA-derived RNAs that contribute to translation inhibition. RNA.

[216] Kompatscher M., Gonnella I., Erlacher M. (2025). Studying the function of tRNA modifications: experimental challenges and opportunities. J. Mol. Biol..

[217] Gupta T., Malkin M.G., Huang S. (2022). tRNA function and dysregulation in cancer. Front. Cell Dev. Biol..

[218] Kapur M., Molumby M.J., Guzman C., Heinz S., Ackerman S.L. (2024). Cell-type-specific expression of tRNAs in the brain regulates cellular homeostasis. Neuron.

[219] Sagi D., Rak R., Gingold H., Adir I., Maayan G., Dahan O. (2016). Tissue- and time-specific expression of otherwise identical tRNA genes. PLoS Genet..

[220] Thomas N.K., Poodari V.C., Jain M., Olsen H.E., Akeson M., Abu-Shumays R.L. (2021). Direct nanopore sequencing of individual full length tRNA strands. ACS Nano.

[221] White L.K., Dobson K., Del Pozo S., Bilodeaux J.M., Andersen S.E., Baldwin A. (2024). Comparative analysis of 43 distinct RNA modifications by nanopore tRNA sequencing. bioRxiv.

[222] Lucas M.C., Pryszcz L.P., Medina R., Milenkovic I., Camacho N., Marchand V. (2024). Quantitative analysis of tRNA abundance and modifications by nanopore RNA sequencing. Nat. Biotechnol..

[223] Shaw E.A., Thomas N.K., Jones J.D., Abu-Shumays R.L., Vaaler A.L., Akeson M. (2024). Combining nanopore direct RNA sequencing with molecular genetics and mass spectrometry for analysis of T-loop base modifications across 42 yeast tRNA isoacceptors. Nucleic Acids Res..

[224] Nakano Y., Gamper H., McGuigan H., Maharjan S., Li J., Sun Z. (2025). Genome-wide profiling of tRNA modifications by Induro-tRNAseq reveals coordinated changes. Nat. Commun..

